# Estimating Trends With Differential Item Functioning: A Comparison of Five IRT-Based Approaches

**DOI:** 10.1177/00131644251408818

**Published:** 2026-03-13

**Authors:** Oskar Engels, Oliver Lüdtke, Alexander Robitzsch

**Affiliations:** 1 IPN–Leibniz Institute for Science and Mathematics Education, Kiel, Germany; 2 Centre for International Student Assessment, Kiel, Germany

**Keywords:** 2PL model, linking, differential item functioning, item parameter drift, regularized estimation, invariance

## Abstract

In longitudinal assessments, tests are frequently used to estimate trends over time. However, when item parameters lack invariance, time-point comparisons can be distorted, necessitating appropriate statistical methods to achieve accurate estimation. This study compares trend estimates using the two-parameter logistic (2PL) model under item parameter drift (IPD) across five trend-estimation approaches for two time points: First, concurrent calibration, which jointly estimates item parameters across multiple time points. Second, fixed calibration, which estimates item parameters at a single time point and fixes them at the other time point. Third, robust linking with Haberman and Haebara as linking methods with $L_{p}$ or $L_{0}$ losses. Fourth, non-invariant items are detected using likelihood-ratio tests or the root mean square deviation statistic with fixed or data-driven cutoffs, and trend estimates are then recomputed using only the detected invariant items under partial invariance. Fifth, regularized estimation under a smooth Bayesian information criterion (SBIC) is applied, shrinking small or null IPD effects toward zero while estimating all others as nonzero. Bias and relative root mean square error (RMSE) were evaluated for the mean and *SD* at T2. An empirical example using synthetic longitudinal reading data, applying the trend-estimation approaches, is provided. The results indicate that the regularized estimation with SBIC performed best across conditions, maintaining low bias and RMSE, followed by robust linking methods. Specifically, Haberman linking with the $L_{0}$ loss function showed superior performance under unbalanced IPD, outperforming the partial invariance approaches. Concurrent and fixed calibration showed the poorest trend recovery under unbalanced IPD conditions.

## Introduction

Longitudinal large-scale assessments (LSAs) in educational and psychological sciences are frequently used to estimate trends over time (Rohm et al., 2021; M. von Davier et al., 2011). These assessments typically employ different but overlapping test forms at successive time points. Examples of such assessments include the National Educational Panel Study (NEPS) in Germany, the Early Childhood Longitudinal Program (ECLS) in the United States, and the National Assessment Program-Literacy and Numeracy (NAPLAN) in Australia (Australian Curriculum, Assessment and Reporting Authority, 2023; Blossfeld et al., 2011; Tourangeau et al., 2018). The overlap created by common items links the time-point-specific forms, thereby aligning the test scores on a common scale (Kolen & Brennan, 2014; A. A. von Davier et al., 2004).

A critical aspect in longitudinal studies and group comparisons is measurement invariance (MI), which indicates that item parameters remain equal (i.e., are invariant) across time points (Meredith, 1993; Millsap, 2011). However, in assessments, MI is often violated (Kreiner & Christensen, 2014; Oliveri & von Davier, 2017; Rohm et al., 2021), leading to differential item functioning (DIF). DIF occurs when individuals from different groups or time points have different probabilities of responding correctly to items, despite possessing equal abilities (Holland & Wainer, 1993; Millsap & Everson, 1993; Penfield & Camilli, 2007). The presence of DIF implies the absence of MI (Borsboom et al., 2008; Millsap, 2011).

In longitudinal comparisons, specifically when item parameters change over time, this phenomenon is called item parameter drift (IPD) and represents a special case of DIF in a temporal context (Goldstein, 1983; Holland & Wainer, 1993). IPD can result from various factors, including shifts in participants’ familiarity with test content, modifications in test administration procedures, or demographic shifts in test-taker populations (Camilli, 1993; Goldstein, 1983; Han et al., 2012; Kolen & Brennan, 2014). This article assumes that IPD may arise from nuisance factors (Shealy & Stout, 1993) or from item-specific heterogeneity over time (van Bork et al., 2024) and considers it a source of bias that should be statistically controlled to ensure valid trend estimation (Kankaraš & Moors, 2014; Kreiner & Christensen, 2014; Oliveri & von Davier, 2017).

In this study, the two-parameter logistic model (2PL; Birnbaum, 1968) is examined exclusively as a special case of a unidimensional item-response theory (IRT) model (van der Linden, 2016). The 2PL model is employed to analyze the relationship between a unidimensional latent trait θ and dichotomous item responses $X_{i}$ for i=1,⋯,I . The item-response function (IRF) for the 2PL model is expressed as

(1) $$P(X_{i}=1|\theta )=P_{i}(\theta ;a_{i},d_{i})=\Psi (a_{i}\theta -d_{i}),$$

where $\Psi (x)=\frac{1}{1+\exp(-x)}$ is the logistic distribution function, and $a_{i}$ and $d_{i}$ are the item discrimination and (negative) item intercept parameters, respectively. We now present IRT estimation for two time points t=1,2 . Let $x_{\mathrm{pt}}=(x_{\mathrm{pt}1},\ldots ,x_{\mathrm{ptI}})$ denote the response vector of person $p=1,\ldots ,N_{t}$ at time point t=1,2 . We define the log-likelihood function for data $D_{t}=(x_{1t},\ldots ,x_{N_{t}t})$ in time point t ( t=1,2 ) as

(2) $$l(\mu_{t},\sigma_{t},a_{t},d_{t};D_{t})=\sum_{p=1}^{N_{t}}\log\left[\int \overset{I}{\underset{i=1}{\Pi }}P_{i}(x_{\mathrm{pti}};\theta ,a_{\mathrm{it}},d_{\mathrm{it}})\phi (\theta ;\mu_{t},\sigma_{t})d\theta \right],$$

where $\phi (\theta ;\mu_{t},\sigma_{t})$ denotes the probability density function of the normal distribution with mean $\mu_{t}$ and standard deviation (*SD*) $\sigma_{t}$ , the vectors of item parameters are defined as $a_{t}=(a_{1t},\ldots ,a_{\mathrm{It}})$ and $d_{t}=(d_{1t},\ldots ,d_{\mathrm{It}})$ , and $P_{i}(x_{\mathrm{pti}};\theta ,a_{\mathrm{it}},d_{\mathrm{it}})$ is the probability of response $x_{\mathrm{pti}}\in \{0,\;1\}$ given by the IRF defined above. The mean and the *SD* of θ at the first time point (T1) are fixed for identification reasons to 0 and 1, respectively.

IPD affecting both discrimination and intercept parameters $a_{\mathrm{it}}$ and $d_{\mathrm{it}}$ is termed nonuniform IPD, while drift affecting only the intercept parameter (with $a_{\mathrm{it}}=a_{i}$ for t=1,2 ) is referred to as uniform IPD (Mellenbergh, 1982). We assume that the 2PL holds for both time points, and that there is uniform IPD, constraining the discrimination parameter $a_{i}$ to be time-invariant (i.e., $a_{i1}=a_{i2}$ ), while allowing the intercept $d_{\mathrm{it}}$ to be time-point-specific

(3) $$P_{\mathrm{it}}(\theta )=\Psi (a_{i}\theta -d_{\mathrm{it}}).$$

An equivalent parameterization of the model uses item difficulties, $b_{\mathrm{it}}$ , which are related to item intercepts by the identity $d_{\mathrm{it}}=a_{i}b_{\mathrm{it}}$ . The model is then written as

(4) $$P_{\mathrm{it}}(\theta )=\Psi (a_{i}(\theta -b_{\mathrm{it}})).$$

In the presence of IPD, the time-point-specific item intercept can be decomposed as

(5) $$d_{\mathrm{it}}=d_{i}+\delta_{\mathrm{it}},$$

where $d_{i}$ represents the time-invariant intercept and $\delta_{\mathrm{it}}$ represents the IPD effect for item i at time t . We focus on a two-time-point design ( T=2 ), for which the general expression reduces to

(6) $$d_{i1}=d_{i}\;\mathrm{and}\;d_{i2}=d_{i}+\delta_{i}\;\mathrm{for}\;i=1,\cdots ,I.$$

This decomposition introduces an identification problem, as the IPD effects $\delta_{i}$ are confounded with the population mean and variance parameters, $\mu_{2}$ and $\sigma_{2}^{2}$ (Bechger & Maris, 2015; Camilli, 1993; Doebler, 2019). Without additional constraints, changes in item parameters cannot be distinguished from changes in the population distribution (Thissen et al., 1993). To address this identifiability issue, we impose a sparsity assumption on the IPD effects $\delta_{i}$ , meaning that the majority of items are assumed to have zero drift, while only a few items are affected (De Boeck, 2008; Frederickx et al., 2010). This sparsity assumption corresponds to the partial invariance assumption (Byrne et al., 1989; Steenkamp & Baumgartner, 1998). Under this assumption, IPD effects can be viewed as outliers that may bias the estimation of trend parameters if not handled appropriately (Chen et al., 2023; De Boeck, 2008; Halpin, 2024; Magis & De Boeck, 2011; W. Wang et al., 2022). We further classify IPD as balanced or unbalanced (González-Betanzos & Abad, 2012; Kopf et al., 2015a). IPD is considered balanced when the IPD effects from Equation (6) sum to zero across the common items C

(7) $$\sum_{i\in C}\delta_{i}=0.$$

However, any choice of weighing of IPD effects can be used to define balanced IPD. Unbalanced IPD occurs when this sum is nonzero (González-Betanzos & Abad, 2012; Kopf et al., 2015a).

The objective of this study is to compare the performance of different trend-estimation approaches for handling sparse uniform IPD across two time points. To this end, we conduct a comparative analysis encompassing five trend-estimation approaches: First, concurrent calibration (CC), which jointly estimates item parameters across multiple time points (e.g., S.-H. Kim & Cohen, 1998; Kolen & Brennan, 2014). Second, fixed calibration (FC), which estimates item parameters at a single time point and fixes them at the other (e.g., S. Kim, 2006; König et al., 2021). Third, robust linking with Haberman (2009; Robitzsch, 2023a) and Haebara as linking methods (Haebara, 1980; He et al., 2015). Fourth, non-invariant items are detected using likelihood-ratio tests (LRTs; e.g., Finch, 2005; Thissen et al., 1988) or the root mean square deviation (RMSD) statistic with fixed (FIX, e.g., Oliveri & von Davier, 2011) or data-driven (DD) cutoffs (M. von Davier & Bezirhan, 2023), and trend estimates are then recomputed using only the detected invariant items under partial invariance. Fifth, regularized estimation (REG) under an smooth Bayesian information criterion is applied, shrinking small or null IPD effects toward zero while estimating all others as nonzero (O’Neill & Burke, 2023; Robitzsch, 2024b).

While individual trend-estimation approaches have been studied under sparse uniform IPD, comprehensive comparisons remain limited. Cho et al. (2016) compared four approaches for handling DIF under the assumption of known DIF items: deleting DIF items, CC under full and partial invariance, and confirmatory multidimensional modeling. However, they did not examine FC, detection-based methods, robust linking approaches, or REG. Finch (2005) compared the LRT against three alternative DIF detection methods using a single significance level and re-estimation via CC only, but they did not examine FC, robust linking methods, or REG. Robitzsch and Lüdtke (2022) compared CC under full and partial invariance, the latter employing RMSD detection with FIX cutoffs, as well as robust Haberman linking (HAB) and Haebara linking (HAE) methods under balanced and unbalanced DIF conditions. Their study, however, did not examine iterative purification approaches, DD RMSD cutoffs (subsequently developed by M. von Davier and Bezirhan, 2023), LRT, FC, or REG. Robitzsch (2023a) compared regularization with the smoothly clipped absolute deviation (SCAD; Fan & Li, 2001) against robust HAB and HAE and RMSD with DD cutoffs under both balanced and unbalanced DIF; however, this study did not examine FC, the LRT, RMSD with FIX cutoffs, or iterative purification. No prior study has compared all five approaches in the specifications employed in this study under both balanced and unbalanced IPD conditions.

The remainder of this article is organized as follows. We introduce five approaches for trend estimation under sparse uniform IPD in the 2PL model. Next, we describe the simulation study design and present the main results. Two additional analyses examine FIX and DD RMSD cutoffs and LRT significance levels. An empirical example using synthetic data from a longitudinal reading comprehension assessment illustrates the application of trend-estimation approaches. Finally, this article concludes with a discussion of the findings and limitations, directions for future research, and a conclusion.

## Approaches for Trend Estimation

In longitudinal assessments with two time points, we distinguish between the following item sets: the set of common items, C , which appear at both time points and serve to link the assessments onto a common scale, the set of unique items, $U_{t}$ , which appear only at time point t (where t=1,2 ), the set of anchor items, A⊆C , which are invariant common items with time-invariant parameters (i.e., $\delta_{i}=0$ for all i∈A ), and the set of biased items, B⊆C , which are non-invariant common items with time-varying parameters (i.e., $\delta_{i}\neq 0$ for t=2 and i∈B ). Note that C=A∪B with A∩B=∅ .

### Concurrent Calibration (CC)

The CC method (e.g., Hanson & Béguin, 2002) estimates parameters for all items at both time points jointly, in a multiple-group IRT model. This model includes both common items C and time-point-specific unique items $U_{t}$ . Common item discriminations a and item intercepts d are estimated by minimizing the estimation function

(8) $$({\hat{\mu }}_{2},{\hat{\sigma }}_{2},\hat{a},\hat{d})=\underset{\left(\mu_{2},\sigma_{2},a,d\right)}{\mathrm{argmin}}\left\{-l(0,1,a,d;D_{1})-l(\mu_{2},\sigma_{2},a,d;D_{2})\right\},$$

with $\mu_{1}$ and $\sigma_{1}$ being fixed for identification reasons to 0 and 1. The CC method enforces parameter invariance for all common items across time points, effectively assuming that C=A and B=∅ . Thus, $\delta_{i}=0$ for all i∈C for t=2 . This assumption is violated when IPD is present, as items that belong to B are incorrectly constrained to have $\delta_{i}=0$ at t=2 . Unique items at each time point are estimated freely within their respective time points. The CC method has been shown to perform well under correct model assumptions and without DIF or IPD (Jodoin et al., 2003; S.-H. Kim & Cohen, 1998; Kolen & Brennan, 2014). When IPD is present, unbalanced IPD typically introduces more bias in trend estimates than balanced IPD, although even balanced IPD can still lead to slightly biased estimates in the 2PL model, as the presence of any IPD may negatively affect the estimation of common item discriminations (Robitzsch, 2023a). The CC method has been extensively studied in various contexts (e.g., Jodoin et al., 2003; S.-H. Kim & Cohen, 1998; Kolen & Brennan, 2014; Lee & Ban, 2009; Robitzsch & Lüdtke, 2022).

### Fixed Calibration (FC)

The FC method (Jodoin et al., 2003; Kang & Petersen, 2012; Keller & Keller, 2011; S. Kim, 2006) is a two-stage procedure. First, item parameters are estimated from the data at the first time point (T1), with the latent trait distribution fixed for identification ( $\mu_{1}=0,\sigma_{1}=1$ ). Here, $a_{1}$ and $d_{1}$ are the vectors of discrimination and intercept parameters for all items at T1 and are estimated as

(9) $$({\hat{a}}_{1},{\hat{d}}_{1})=\underset{\left(a_{1},d_{1}\right)}{\mathrm{argmin}}\left\{-l(0,1,a_{1},d_{1};D_{1})\right\}.$$

Second, the estimated item parameters ${\hat{a}}_{i1}$ and ${\hat{d}}_{i1}$ at T1 for common items, i∈C , are held fixed when fitting the model to data from the second time point (T2). These fixed values serve as equality constraints for the T2 calibration

(10) $$({\hat{\mu }}_{2},{\hat{\sigma }}_{2},\{{\hat{a}}_{i2},{\hat{d}}_{i2}{\}}_{i\in U_{2}})=\underset{\begin{array}{c} (\mu_{2},\sigma_{2}),\\ {\left\{a_{i2},d_{i2}\right\}}_{i\in U_{2}} \end{array}}{\mathrm{argmin}}\left\{-l(\mu_{2},\sigma_{2},a_{2},d_{2};D_{2})\right\},$$

where $a_{2}$ and $d_{2}$ denote the combined parameter vectors, with the constraint that $a_{i2}={\hat{a}}_{i1}$ and $d_{i2}={\hat{d}}_{i1}$ for all common items i∈C , while parameters for unique items $i\in U_{2}$ are freely estimated. With no unique items present at T2, this simplifies to

(11) $$({\hat{\mu }}_{2},{\hat{\sigma }}_{2})=\underset{\left(\mu_{2},\sigma_{2}\right)}{\mathrm{argmin}}\left\{-l(\mu_{2},\sigma_{2},{\hat{a}}_{1},{\hat{d}}_{1};D_{2})\right\},$$

where only the distribution parameters ( $\mu_{2},\sigma_{2}$ ) are freely estimated at T2. Like CC, this method assumes that all common items are invariant ( C=A , B=∅ ). FC has been found to perform satisfactorily under no IPD, or under partial invariance when items with detected drift are excluded from the common item set before calibration (Hu et al., 2008; König et al., 2021). In the presence of DIF, FC yields biased estimates of the mean (e.g., Sachse et al., 2016), and there is also evidence for bias in the *SD* (e.g., Robitzsch, 2024a). The bias in the estimated mean can change sign when ability distributions differ across administrations (Keller & Keller, 2011).

### Robust Linking

Robust linking is a two-step process. First, item parameters are calibrated separately for both time points, without invariance constraints, typically with identification constraints $\mu_{t}=0$ and $\sigma_{t}=1$ . In the second step, robust linking methods place these separately estimated parameters onto a common scale using the common items C . In robust linking methods, the sets A and B are determined implicitly by down-weighting outlier items. T1 serves as the reference scale, and two linking constants, A and B , transform T2 to this scale via $\theta_{2}^{*}=A\theta_{2}+B$ , such that $\theta_{2}^{*}\sim N(B,A^{2})$ . The linking constant A represents the estimated *SD* ${\hat{\sigma }}_{2}$ , whereas B denotes the estimated mean ${\hat{\mu }}_{2}$ for T2. Non-robust linking uses the $L_{2}$ loss function, while robust methods employ loss functions that minimize the influence of items with IPD when determining linking constants, effectively down-weighting biased items. The choice of the loss function is central to the robustness of these methods. This study focuses on the versatile family of $L_{p}$ loss functions and the related $L_{0}$ loss function, which are described next.

#### *L_p_* and *L_0_* Loss Functions

The $L_{p}$ loss function family (Lipovetsky, 2007) is defined as

(12) $$\rho (x)=|x{|}^{p}\;\mathrm{for}\;p>0.$$

The $L_{2}$ loss corresponds to squared loss, while $L_{1}$ corresponds to median regression (Koenker, 2017; Koenker & Hallock, 2001). For p≤1 , the function is non-differentiable at x=0 . A differentiable approximation of ρ can be used as

(13) $$\tilde{\rho }(x)=(|x{|}^{2}+\varepsilon {)}^{p/2},$$

where ε>0 is a tuning parameter that controls the approximation error of $\tilde{\rho }$ (see Asparouhov & Muthén, 2014). Values of ε=0.01 (Asparouhov & Muthén, 2014) and ε=0.001 (Robitzsch, 2025b) have shown satisfactory performance. An alternative to the $L_{p}$ loss function is the $L_{0}$ loss function (Oelker et al., 2015; Oelker & Tutz, 2017), which indicates a deviation from zero and is defined as

(14) ρ(x)=1(x≠0).

An approximation of this loss function can be found in O’Neill and Burke (2023)

(15) $$\tilde{\rho }(x)\approx \frac{x^{2}}{x^{2}+\varepsilon }.$$

The approximation of the $L_{0}$ loss function has been shown to outperform approximations of other $L_{p}$ loss functions for small values of p (Robitzsch, 2023b). A value of ε=0.01 has been shown to perform well in various settings and will be applied in this study for the $L_{0}$ approximation with p=0 (e.g., Robitzsch, 2025b). The $L_{p}$ approximation will be used with p>0 and ε=0.001 .

#### Haberman Linking (HAB)

Haberman (2009) introduced a regression technique that extends the mean-geometric mean (MGM) method for multiple time points. The original Haberman formulation uses the $L_{2}$ loss. The regression model uses log-transformed item discriminations and item intercepts, where the mean and *SD* of T1 are set to 0 and 1, respectively, for identification. First, the log-transformed *SD* of T2, $s_{2}$ , and the common logarithmized item discriminations $\kappa =(\kappa_{i},\cdots ,\kappa_{I})$ , are estimated as

(16) $$(\hat{s_{2}},\hat{\kappa })=\underset{\left(s_{2},\kappa \right)}{\mathrm{argmin}}\left\{\sum_{i\in C}\rho (\log{\hat{a}}_{i1}-\kappa_{i})+\sum_{i\in C}\rho (\log{\hat{a}}_{i2}-s_{2}-\kappa_{i})\right\},$$

where ρ is the $L_{p}$ or $L_{0}$ loss function. The untransformed *SD* of the T2 is obtained as ${\hat{\sigma }}_{2}=\exp({\hat{s}}_{2})$ . Under uniform IPD, where $a_{i}$ remains invariant across time points, this *SD* estimation remains unaffected by the drift in intercept parameters. Second, the mean $\mu_{2}$ can be estimated based on either item difficulties ${\hat{b}}_{\mathrm{it}}$ or item intercepts ${\hat{d}}_{\mathrm{it}}$ . The former is the original version proposed by Haberman (2009). The common item difficulties $b=(b_{i},\cdots ,b_{I})$ are estimated as

(17) $$({\hat{\mu }}_{2},\hat{b})=\underset{\left(\mu_{2},b\right)}{\mathrm{argmin}}\left\{\sum_{i\in C}\rho ({\hat{b}}_{i1}-b_{i})+\sum_{i\in C}\rho \left({\hat{\sigma }}_{2}{\hat{b}}_{i2}+\mu_{2}-b_{i}\right)\right\}.$$

Estimation based on item intercepts is performed as (Robitzsch, 2025a)

(18) $$({\hat{\mu }}_{2},\hat{d})=\underset{\left(\mu_{2},d\right)}{\mathrm{argmin}}\left\{\sum_{i\in C}\rho ({\hat{d}}_{i1}-d_{i})+\sum_{i\in C}\rho \left({\hat{d}}_{i2}+\frac{{\hat{a}}_{i2}}{\sigma_{2}}\mu_{2}-d_{i}\right)\right\}.$$

The intercept parameterization yields more precise trend estimates due to the lower estimation variance of intercept parameters compared to difficulty parameters (Robitzsch, 2025a). Unlike MGM linking, which directly uses group-specific item parameter estimates, HAB simultaneously estimates joint item parameters across both groups. Empirical evidence from Robitzsch (2025b) indicates modest efficiency gains from this approach in two-group settings, even though it was originally proposed and used for linking multiple groups or time points.

#### Haebara Linking (HAE)

HAE linking (Haebara, 1980) minimizes the discrepancy between the IRFs based on item parameters obtained from separate calibrations. The linking function, based on the item difficulty parameterization ( b=d/a ), is defined as

(19) $$({\hat{\mu }}_{2},{\hat{\sigma }}_{2})=\underset{\left(\mu_{2},\sigma_{2}\right)}{\mathrm{argmin}}\{\sum_{i\in C}\int \rho (\Psi ({\hat{a}}_{i1}[\sigma_{2}\theta +\mu_{2}-{\hat{b}}_{i1}])-\Psi ({\hat{a}}_{i2}[\theta -{\hat{b}}_{i2}]))\omega (\theta )d\theta \},$$

where ρ is the $L_{p}$ or $L_{0}$ loss function, and ω is a weighting function. The weighting function can be uniform or a normal-density function. Haebara’s original proposal used the empirical frequency of ability estimates as weights, which is closely approximated by a normal-density function (Haebara, 1980; Robitzsch, 2025c). A recent variant, called information-weighted HAE, weights the squared deviations by the sum of the item information functions from both groups to reduce the impact of parameter estimation errors (S. Wang et al., 2024; W. Wang et al., 2022). However, simulations by Robitzsch (2025c) found that, while this approach outperforms HAE with uniform weights, it is inferior to normal-density weights in terms of bias and root mean square error (RMSE). Normal-density weights emphasize the ability scale center, where estimates are more precise, thereby reducing estimation error influence from the tails and decreasing linking constant variance (Robitzsch, 2025c). In contrast to HAB, HAE simultaneously estimates both $\mu_{2}$ and $\sigma_{2}$ by minimizing differences between the IRFs [Equation (19)]. Therefore, uniform IPD affecting item intercepts influences both parameter estimates. The original Haebara method employed an $L_{2}$ loss ( p=2 ), which is sensitive to outliers. To increase robustness against IPD, variations using an $L_{1}$ loss ( p=1 ) were proposed (He & Cui, 2019; He et al., 2015). Further research demonstrated that even smaller exponents ( p<1 ) can reduce bias more effectively in the presence of unbalanced IPD (Robitzsch, 2020). This increased robustness was found to result in only a small loss of statistical efficiency (i.e., a higher RMSE) in scenarios involving no or balanced IPD (Robitzsch, 2020; Robitzsch & Lüdtke, 2022). In addition, it should be noted that the standard HAE procedure is asymmetric, aligning the IRFs from T1 onto those of T2. This asymmetry implies that the direction of linking can influence the results when IPD is present, as the method minimizes deviations in only one direction. For a symmetric HAE method, see S. Kim and Kolen (2007), Arai and Mayekawa (2011), and Weeks (2010).

### Partial Invariance Using IPD Statistics

Partial invariance using IPD statistics handles non-invariance in a two-stage procedure. First, items exhibiting IPD are detected using IPD statistics. Then, they are accounted for in a subsequent modeling step (e.g., Penfield & Camilli, 2007; Wu, 2010). This approach partitions the set of common items C into sets A and B . After identifying the biased items, a model under partial invariance is re-estimated. In this model, the item parameters of the items in B are estimated freely across time points, while item parameters in the anchor set A are constrained to be equal. The trend is then estimated from the final partial invariance model. In this model, the common scale is established by the anchor items (Robitzsch & Lüdtke, 2022).

The detection-based approach faces the circular problem of needing a set of DIF-free items to reliably detect items with DIF or IPD (Angoff, 1982; Doebler, 2019). If the initial anchor set contains biased items, it can distort the detection process and inflate Type I error rates for other items (Shaffer, 1995). While various IPD statistics exist in the literature (see Penfield & Camilli, 2007, for an overview), this study focuses on the significance-based LRT (Thissen et al., 1988) and the effect-size-based RMSD (Tijmstra et al., 2020), using FIX and DD cutoffs (M. von Davier & Bezirhan, 2023).

#### Re-estimation and Item Purification

A variety of approaches exist for implementing IPD detection methods (e.g., Kopf et al., 2015a). In this study, a one-step (OS) approach and a forward-only iterative (IT) approach are utilized for RMSD (Magis et al., 2010). In this study, we implement both the OS and IT approaches for RMSD. In contrast, the LRT is evaluated using only the OS approach due to computational constraints that require k+1 model calibrations per iteration, where k is the number of items tested.

In the OS approach, all items in C are initially assumed invariant. IPD statistics are computed for each item i∈C , and items exceeding the threshold are reassigned from A to B . The procedure terminates here or continues iteratively. In the IT approach, items that are flagged as non-invariant are not retested in later iterations. Each iteration re-estimates a model under partial invariance, freeing parameters for items in B and maintaining equality constraints for items in A , and recomputes RMSD statistics only for the items that remain in A . Newly flagged items are transferred to B , and the cycle repeats until no additional items are flagged or a maximum set of iterations is reached. Similar to the implementation of the difR package (Magis et al., 2010), we set a maximum number of iterations. While the difR authors chose 10 iterations, we opted for 7 iterations.

After IPD detection identifies biased items, the resulting anchor set A is used for trend estimation through two approaches: CC under partial invariance, which frees time-point-specific parameters for items in B while maintaining equality constraints for A , and linking methods applied to the anchor items. For linking, we use the squared loss ( p=2 ) for HAB (with item intercepts and item difficulties) and HAE (with uniform and normal densities, with σ=0.5,1, and 2 ).

The IT approach carries inherent risks. As items are removed, remaining anchors bear greater responsibility for scale identification. Type I errors can create a cascade effect where contaminated anchors lead to further misclassifications (e.g., Kopf et al., 2015b). Early false positives cannot be corrected in later iterations.

To ensure model identifiability across LRT and RMSD, a minimum of three common items must remain in the anchor set A . Two items would theoretically suffice for identification in the 2PL model; three anchor items provide more stable trend estimation and reduce sensitivity to parameter estimation errors in individual items. If detection procedures flag more items than this constraint allows, only those with the highest $\chi^{2}$ values for LRT, or the highest FIX or DD RMSD values, are flagged, up to |C|−3 items. If no items are flagged, the re-estimation proceeds with C=A .

#### Root Mean Square Deviation (RMSD)

The RMSD for a common item i∈C at time point t assesses the distance between a time-point-specific IRF $P_{\mathrm{it}}$ and the model-implied IRF under invariance $P_{i}$ (Thissen et al., 1988). It is calculated as

(20) $${\mathrm{RMSD}}_{\mathrm{it}}=\sqrt{\int {\left(P_{\mathrm{it}}(\theta )-P_{i}(\theta )\right)}^{2}f_{t}(\theta )d\theta },$$

where $P_{i}(\theta )$ represents the IRF under the invariance constraint (using the pooled or constrained parameter estimates across time points as defined earlier), and $f_{t}$ is the density of θ at time point t . It is important to note that the sample-based RMSD is a biased estimator of its population counterpart. Therefore, it tends to be positively biased in smaller samples due to sampling variability (Köhler et al., 2020). Its value is context-dependent, as item misfit can inflate RMSD values for well-fitting items in the same test (M. von Davier & Bezirhan, 2023). For more details on estimating the RMSD, see Köhler et al. (2020) and Tijmstra et al. (2020).

##### Fixed Cutoffs

Items are flagged when RMSD exceeds a FIX cutoff. Proposed cutoffs in the literature range from 0.05 (Robitzsch & Lüdtke, 2022) to 0.20 (OECD, 2016). Simulation studies demonstrated that stricter cutoffs (0.05, 0.08) outperform lenient thresholds in controlling bias and identifying biased items (Buchholz & Hartig, 2019; Fährmann et al., 2022; Köhler et al., 2020; Robitzsch & Lüdtke, 2022). Based on this evidence, this study employs a range of strict to moderate cutoffs (0.03, 0.05, and 0.08) to evaluate their effectiveness.

##### Data-Driven Cutoffs

The DD approach proposed by M. von Davier and Bezirhan (2023) offers an alternative for identifying items with IPD effects using median-based statistics. Unlike FIX cutoffs, which apply predetermined cutoffs regardless of the data distribution, this approach derives cutoffs DD from the observed RMSD distribution itself. First, the median absolute deviation (MAD) of the RMSD values is computed as the median of the absolute deviations from the median RMSD

(21) $$\mathrm{MAD}={\mathrm{median}}_{i\in C}(|{\mathrm{RMSD}}_{i}-{\mathrm{median}}_{j\in C}({\mathrm{RMSD}}_{j})|),$$

where ${\mathrm{RMSD}}_{i}$ denotes the value for item i , and the index j serves as a running index over all common items when computing the inner median. A robust z-score is then calculated for each common item i∈C at time point t

(22) $$z_{i}=\frac{\left|{\mathrm{RMSD}}_{i}-{\mathrm{median}}_{j\in C}({\mathrm{RMSD}}_{j})\right|}{1.4826\times \mathrm{MAD}},$$

where the scaling constant 1.4826 makes the MAD comparable to the *SD* under normality assumptions (Huber, 1981). We estimated the MAD separately for T1 and T2.

Items are flagged as exhibiting IPD if their robust z-score exceeds a critical value $|z_{i}|\;>\;\tau$ . Since the RMSD has a meaningful lower bound of zero, items are flagged only if their robust z-score exceeds the positive cutoff, effectively making it a one-sided test for large deviations (M. von Davier & Bezirhan, 2023). M. von Davier and Bezirhan (2023) evaluated cutoffs of 2, 2.5, and 3 in their original study. The present study examines two cutoff levels, τ=1.7 and τ=2.7 , to evaluate detection performance across different stringencies. The value of 2.7 has been employed in subsequent applications (Robitzsch, 2023a) and aligns with robust outlier detection practices in IRT models (Huynh & Meyer, 2010; Liu & Jurich, 2022). The DD approach adapts to the empirical RMSD distribution, making it less sensitive to sample-specific peculiarities than FIX cutoffs. M. von Davier and Bezirhan (2023) found that DD was more sensitive than a relatively lenient FIX cutoff of 0.15. However, stricter FIX cutoffs (e.g., 0.05 and 0.08) have been shown to identify IPD items better and reduce bias in trend estimates (Buchholz & Hartig, 2019; Fährmann et al., 2022; Köhler et al., 2020; Robitzsch & Lüdtke, 2022). Accordingly, the present study compares DD alongside these lower FIX cutoffs.

#### Likelihood-Ratio Test (LRT)

The LRT for IPD detection (Thissen et al., 1988) compares nested IRT models. This study employs the constrained-baseline model approach, also known as the all-other anchor method, which is common in IRT research (Stark et al., 2006; W.-C. Wang & Yeh, 2003). In this approach, the baseline model (Model 0) constrains all common items to be invariant ( $a_{i1}=a_{i2}=a_{i}$ , and $d_{i1}=d_{i2}=d_{i}$ for all i∈C ). To test a specific item for IPD, an alternative model (Model 1) is fitted, in which the item’s parameters are freely estimated across time points. At the same time, the invariance constraints are maintained for all other common items, which serve as the anchor set that establishes the common metric (Cohen et al., 1996; S. H. Kim & Cohen, 1995). The test statistic is computed as

(23) $$G^{2}=-2(\log\;L_{0}-\log\;L_{1}),$$

where $L_{0}$ is the likelihood of the baseline model (Model 0), which constrains the item to be invariant, and $L_{1}$ is the likelihood of the alternative model (Model 1), where the item’s parameters are freely estimated. The statistic follows a chi-square distribution with df=2 for the 2PL model (S. H. Kim & Cohen, 1995). Items are flagged when $G^{2}$ exceeds the critical value corresponding to the chosen significance level. We apply α=0.05 , α = 0.01, and Bonferroni-corrected α=0.05/k , where k is the number of items tested (Bonferroni, 1936). The LRT procedure is computationally demanding, requiring k+1 separate model calibrations for a test involving k items (S. H. Kim & Cohen, 1995).

Under no IPD for the 2PL model, the LRT maintains Type I error rates close to the nominal alpha level (Cohen et al., 1996; S.-H. Kim & Cohen, 1998). While standard alpha levels (e.g., α=0.05 ) can lead to inflated Type I error rates when the anchor set is contaminated (Finch, 2005; González-Betanzos & Abad, 2012; Stark et al., 2006), stricter Bonferroni corrections reduce statistical power with small sample sizes or small IPD effects (Stark et al., 2006). Anchor set quality crucially influences LRT power, with higher-discrimination anchors improving detection rates (Lopez Rivas et al., 2009). Unbalanced IPD poses problems for the constrained-baseline approach by distorting the latent scale (W. Wang et al., 2022).

### Regularized Estimation (REG)

Regularization methods handle IPD by addressing the model identification problem directly within the estimation framework. If IPD effects $\delta_{i}$ were freely estimated for all items in C , the model would be unidentified, as the item-level drift parameters would be perfectly confounded with the overall trend parameters ( $\mu_{t},\sigma_{t}$ ) (Bechger & Maris, 2015; Doebler, 2019). Unlike CC, which enforces full invariance by assuming B=∅ (all $\delta_{i}=0$ ), or detection methods that explicitly partition C into A and B , regularization treats all items in C as potentially having IPD under a sparsity assumption. Therefore, in Equation (5), most items are expected to have $\delta_{i}=0$ , while only a subset have nonzero effects.

The REG approach employs a multiple-group IRT framework, where both time points are estimated simultaneously. For time points t=1,2 , the negative log-likelihood function is

(24) $$l^{*}(\mu_{2},\sigma_{2},a,d,\delta )=-\sum_{t=1}^{2}\sum_{p=1}^{N_{t}}\log\left[\int \overset{I}{\underset{i=1}{\Pi }}P_{\mathrm{it}}(x_{\mathrm{pti}};\theta ,a_{i},d_{\mathrm{it}})\phi (\theta ;\mu_{t},\sigma_{t})d\theta \right],$$

with $\mu_{1}=0$ and $\sigma_{1}=1$ fixed for identification, $a=(a_{1},\ldots ,a_{I})$ contains the time-invariant discrimination parameters, $d=(d_{1},\ldots ,d_{I})$ contains the baseline intercept parameters, and $\delta =(\delta_{1},\ldots ,\delta_{I})$ contains the IPD effects. Note that $d_{\mathrm{it}}\;=\;d_{i}\;+\;\delta_{\mathrm{it}}$ , as defined in Equation (5), where $\delta_{i1}=0$ and $\delta_{i2}=\delta_{i}$ for the two-time-point case. Compared to Equation (2), the parameter vector now contains $\delta =(\delta_{1},\cdots ,\delta_{I})$ . This overidentified model with all potential IPD effects is made identifiable by adding a penalty term P to the log-likelihood function, and the REG problem becomes

(25) $$({\hat{\mu }}_{2},{\hat{\sigma }}_{2},\hat{a},\hat{d},\hat{\delta })=\underset{\left(\mu_{2},\sigma_{2},a,d,\delta \right)}{\mathrm{argmin}}\left\{l^{*}(\mu_{2},\sigma_{2},a,d,\delta )+N^{*}\sum_{i\in C}P(\delta_{i},\lambda )\right\},$$

where $N^{*}=N_{1}+N_{2}$ is the total sample size across both time points, and $P(\delta_{i},\lambda )$ is a penalty function with regularization parameter λ .

While penalty functions such as the least absolute shrinkage and selection operator (LASSO; Tibshirani, 1996) and the SCAD (Fan & Li, 2001) could be applied, these require computationally intensive grid search or cross-validation to select optimal λ values, making them less efficient for large-scale assessment applications (Robitzsch, 2024b). In addition, the LASSO is known to produce biased estimates for nonzero coefficients and therefore underestimates the magnitude of true IPD effects (Fan & Li, 2001). Previous research has shown that regularization based on a grid search does not always perform convincingly in the pure recovery of population parameters compared to other approaches, such as robust linking (see Robitzsch, 2023a). This study, therefore, employs a more recent regularization estimation approach proposed by O’Neill and Burke (2023), which directly optimizes the smooth Bayesian information criterion (SBIC). This method avoids the computational burden of grid search and has demonstrated the ability to accurately recover population parameters in the context of IRT models under DIF and was found to be the best-performing regularization method for estimating group means in the presence of unbalanced DIF (Robitzsch, 2024b).

The SBIC method modifies the estimation approach based on the Bayesian information criterion (BIC). While the BIC for a regularized model penalizes complexity by counting the number of nonzero parameters using a non-differentiable indicator function, $1(\delta_{i}\neq 0)$ , the SBIC approach replaces this discrete counter with a smooth, differentiable approximation that allows for direct optimization (O’Neill & Burke, 2023; Robitzsch, 2024b)

(26) $$N_{\varepsilon }(x)=\frac{x^{2}}{x^{2}+\varepsilon },$$

where ε>0 is a small tuning parameter. The resulting SBIC to be minimized is

(27) $$\mathrm{SBIC}(\mu_{2},\sigma_{2},a,d,\delta )=2l^{*}(\mu_{2},\sigma_{2},a,d,\delta )+\log(N^{*})\left(H+\sum_{i\in C}N_{\varepsilon }(\delta_{i})\right),$$

where H is the number of non-penalized parameters, and the sum runs over all potentially non-invariant IPD effects. We use three values of the tuning parameter ε=0.01,0.001, and 0.0001 , while Robitzsch (2024b) found ε=0.001 to perform best. The alternative smooth Akaike information criterion (SAIC) was found to have higher Type I error rates. It was outperformed by SBIC in parameter recovery, supporting the use of the SBIC for this application (Robitzsch, 2024b). The final parameter estimate is obtained by

(28) $$({\hat{\mu }}_{2},{\hat{\sigma }}_{2},\hat{a},\hat{d},\hat{\delta })=\underset{\left(\mu_{2},\sigma_{2},a,d,\delta \right)}{\mathrm{argmin}}\mathrm{SBIC}(\mu_{2},\sigma_{2},a,d,\delta ).$$

The direct optimization of this criterion performs the estimation of all model parameters and the selection of items with nonzero IPD effects simultaneously. In this integrated process, the IPD effects ( $\delta_{i}$ ) of items that are functionally invariant are shrunk toward zero, implicitly defining the set of anchor items A within the single estimation step.

## Simulation Study

### Purpose

This simulation study assesses the performance of five trend-estimation approaches, as described in section “Approaches for Trend Estimation,” in the 2PL model when item parameters are affected by sparse uniform IPD across two time points. The primary goal is to investigate how these trend-estimation approaches perform under varying conditions, particularly the distinction between balanced and unbalanced IPD. The latter is known to challenge methods that assume full invariance or do not employ robust estimation techniques (DeMars, 2019; Robitzsch & Lüdtke, 2022). Therefore, we investigate the extent to which the factors of balanced and unbalanced IPD, the magnitude of IPD effects, the proportion of affected items, sample size, and the number of items influence the accuracy of trend estimates, in terms of bias and root mean square error. We examine contamination scenarios with 10% and 30% affected items, and include a larger mean shift ( $\mu_{2}=1.0$ ) to assess performance under a larger developmental change.

Under no IPD conditions, we expect CC to provide unbiased estimates with the lowest RMSE (Hanson & Béguin, 2002; Jodoin et al., 2003; S.-H. Kim & Cohen, 1998; Kolen & Brennan, 2014; Robitzsch & Lüdtke, 2022). FC should also yield unbiased performance under these ideal conditions (Hu et al., 2008; König et al., 2021). Robust linking methods are anticipated to show a slight efficiency loss compared to CC due to unnecessary robustification when no IPD is present (He & Cui, 2019; He et al., 2015; Robitzsch, 2023a; Robitzsch & Lüdtke, 2022). The detection-based methods should maintain nominal error rates, although they may experience some efficiency loss due to sampling variability in the detection process (Cohen et al., 1996; Finch, 2005; S. H. Kim & Cohen, 1995). The REG is expected to produce unbiased estimates with minimal shrinkage effects when no items exhibit drift (Robitzsch, 2024b).

For balanced IPD conditions, we anticipate that CC, and, to a lesser extent FC, will remain largely unbiased, mitigated by the cancelation effects of symmetric IPD patterns (Chalmers et al., 2015), although slight bias may occur in CC (Hanson & Béguin, 2002; Kolen & Brennan, 2014), and FC is expected to produce biased *SD* estimates due to model misspecification and the mean shift between T1 and T2 (Robitzsch, 2024a). Robust linking methods with p≤1 are expected to show reduced bias compared with the $L_{2}$ loss function (He & Cui, 2019; Robitzsch, 2023a), with HAB based on intercepts being more efficient than HAB based on difficulties (Robitzsch, 2025a). The performance of the detection method will depend critically on correct item identification, with LRT being particularly susceptible to contaminated anchor set effects (Cohen et al., 1996; Stark et al., 2006; W.-C. Wang & Yeh, 2003). REG is expected to maintain unbiased estimation through selective shrinkage of IPD effects (Robitzsch, 2024b). However, the comparative performance of REG versus robust linking methods under balanced IPD with lower IPD item percentages (10% and 30%) remains an open question.

Under unbalanced IPD conditions, we expect the most severe challenges for trend estimation (Chalmers et al., 2015). CC and FC should exhibit unsatisfactory bias that does not diminish with increasing sample size, reflecting fundamental model misspecification (DeMars, 2019; Robitzsch & Lüdtke, 2022). Among robust linking methods, the $L_{0}$ loss function is expected to minimize bias most effectively, while normal-density weights for HAE are expected to improve precision relative to uniform weights (Robitzsch, 2025b, 2025c). The LRT will face a trade-off between Type I error rates when using the Bonferroni correction and statistical power when using standard significance levels (Finch, 2005; González-Betanzos & Abad, 2012; Stark et al., 2006). For RMSD-based detection, DD cutoffs outperformed a FIX cutoff of 0.15, but it is still uncertain how they compare to lower FIX cutoffs (M. von Davier & Bezirhan, 2023). REG is anticipated to provide strong performance under unbalanced IPD, particularly with the tuning parameter ε=0.001 (Robitzsch, 2024b). However, the comparative advantage of REG versus robust linking under unbalanced IPD remains to be seen. Finally, we aim to identify which method specifications yield optimal performance across diverse conditions and, in which IPD conditions, the CC and FC methods, which are misspecified, yield unbiased results.

### Method

For the analysis, the ability variable θ for T1 was assumed to follow a standard normal distribution (i.e., $\theta_{1}\sim N(0,\;1)$ ). For T2, the mean was set to $\mu_{2}=1.0$ , and the *SD* was set to $\sigma_{2}=1.3$ . The increased mean represents a larger growth between measurement occasions, while the increased *SD* reflects the assumption that individual growth trajectories diverge over time. The simulation employed a fixed set of 10 base items, with item discrimination parameters $a_{i}$ being 1.06, 0.78, 0.91, 1.14, 1.19, 0.89, 0.82, 1.00, 1.00, and 1.00, with values ranging from 0.78 to 1.19 (*M* = 0.98, *SD* = 0.12), and item intercept parameters $d_{i}$ were −0.17, −0.77, 0.36, 1.37, 2.08, −1.56, 0.72, −0.46, −0.46, and −0.46, ranging from −1.56 to 2.08 (*M* = 0.07, *SD* = 1.12). The discrimination parameters for IPD-affected items (Items 8, 9, and 10 in each block) were set to $a_{i}=1.0$ to simplify interpretation and ensure comparability with previous studies that used difficulty-based data generation (Robitzsch, 2023a; M. von Davier & Bezirhan, 2023). In addition, using the base item set avoids confounding with simulation factors (e.g., test length). The item parameters can also be found at https://osf.io/q86jz. All items were treated as common items across both time points ( C=I and $U_{t}=\;\emptyset$ ).

The simulation design was configured to vary five factors: (a) The sample size ( N ) was set at 500, 1,000, and 2,500 for each time point, representing typical sample sizes commonly employed in practice and methodological research (e.g., Berrío et al., 2020; Harwell et al., 1996). (b) The number of items ( I ) was set at either 20 or 40, obtained by duplicating or quadruplicating the base set of 10 items (i.e., using two or four 10-item blocks). (c) The IPD effect size ( δ ) was set to 0, indicating an absence of IPD, 0.5, indicating moderate IPD, or 1.0, indicating large IPD, for the item intercept $d_{i}$ on designated IPD items. (d) The percentage of common items affected by IPD (%IPD) was 0%, 10%, or 30%. For 10% IPD, Item 10 in each 10-item block exhibited IPD. For 30% IPD, Items 8, 9, and 10 in each 10-item block were affected, all with $a_{i}=1.00$ . (e) The IPD type was balanced, in which positive and negative effects averaged to zero across affected items (half received $\delta_{i}=|\delta |$ , and half received $\delta_{i}=-|\delta |$ ), or unbalanced, in which all affected items received negative effects ( $\delta_{i}=-|\delta |$ ), uniformly decreasing the item intercepts at T2. Dichotomous item responses were generated according to a 2PL model for each time point. At T2, uniform sparse IPD effects were applied according to Equation (6) by adding $\delta_{i}$ to the base intercept parameter $d_{i}$ for the specified items. After eliminating redundant conditions in which no IPD would be present (i.e., conditions with either δ=0 or %IPD = 0%, but not both), the design yielded 54 unique simulation conditions: 3 sample sizes ×2 item counts× ([2 IPD effect sizes ×2 IPD percentages ×2 IPD types] + 1 no-IPD condition). A total of 1,000 replications were conducted for each condition.

The study compared five approaches of trend-estimation methods (described in section “Approaches for Trend Estimation”). For CC, time points T1 and T2 were calibrated simultaneously in a multiple-group 2PL model. FC used the estimated item parameters from T1 as fixed values when calibrating T2. The robust linking methods included the HAB and HAE approaches with $L_{p}$ loss functions for p=.25,.5,1, and 2 , as well as the $L_{0}$ loss function. The HAB method used item intercepts and item difficulties, whereas the HAE method used four weighting functions: uniform weighting, and normal-density weighting with σ=0.5,1, and 2 . The detection-based methods employed the LRT with α=0.05,0.01 , and the Bonferroni correction ( α=0.05 ) with the OS approach. The RMSD was used with FIX cutoffs (0.03, 0.05, and 0.08) and DD cutoffs ( τ=1.7 and 2.7 ) in both the OS and IT approaches. For re-estimation after detection, we applied CC, HAB (with item intercepts or item difficulties), and HAE (with the aforementioned four weighting functions), all with the $L_{2}$ loss function. An IT approach with a maximum of 7 iterations was implemented for RMSD methods. REG employed SBIC with ε=0.01,0.001, and 0.0001 . In total, the combinations of all the previously described methods and their specifications resulted in 126 distinct trend estimators.

To present the main findings, we selected the best-performing specification for each approach based on absolute bias in the most challenging condition: unbalanced IPD, 30% IPD, δ=1.0 , I=20 , and N=2,500 . The sample size was chosen for asymptotic assessment (the largest sample size, N=2,500 ). The complete results for this condition are reported in the Supplement (Table S1). The selected specifications are as follows: We used REG with SBIC ( ε=0.001 ). For robust linking, we chose HAB with item intercepts and HAE with normal-density weighting ( σ=0.5 ). For detection-based estimation, we employed the LRT with a Bonferroni correction ( α=0.05 ), followed by re-estimation using HAB with item difficulties. Regarding the RMSD, we considered two variants: a FIX cutoff of 0.05 using the IT approach, followed by HAE with normal-density weighting ( σ=1 ); and a DD cutoff with τ=1.7 using the IT approach, followed by HAE with normal-density weighting ( σ=0.5 ). After reporting the results for these specifications, we present two additional results within the simulation study. The first examines how RMSD FIX cutoffs (0.03, 0.05, and 0.08) and DD cutoffs ( τ=1.7 and 2.7 ), as well as OS versus IT approaches, affect trend recovery. The second set of additional results evaluates how different LRT significance levels influence the re-estimated trends.

The performance of each method was assessed based on the recovery of the two trend parameters of interest: the mean $\mu_{2}$ and the *SD* $\sigma_{2}$ . For each simulation condition with R replications ( r=1,⋯,R ), the parameter estimate was ${\hat{\vartheta }}_{r}$ (either ${\hat{\mu }}_{2r}$ or ${\hat{\sigma }}_{2r}$ ). The bias of an estimated parameter was calculated as

(29) $$\mathrm{Bias}(\hat{\vartheta })=\frac{1}{R}\sum_{r=1}^{R}({\hat{\vartheta }}_{r}-\vartheta ).$$

The RMSE was estimated by

(30) $$\mathrm{RMSE}(\hat{\vartheta })=\sqrt{\frac{1}{R}\sum_{r=1}^{R}{\left({\hat{\vartheta }}_{r}-\vartheta \right)}^{2}}.$$

To enhance comparability across sample sizes, the RMSE was normalized against the REG approach, which was set as the reference method with a value of 100 in each condition. The REG approach was selected as the reference method because it aligns with the data-generating model used in the simulation study. It assumes sparse uniform IPD; therefore, only the item intercepts have IPD effects ( $\delta_{i}$ ) at T2, while item discriminations remain invariant across time points. An estimator was considered satisfactory if its relative RMSE was 125 or less, indicating that its RMSE was no more than 25% higher than that of the reference method. Performance for bias was considered satisfactory if the absolute bias was less than 0.015. This threshold was determined based on considerations for two-sample comparisons with $\sigma^{2}=1$ . For N=2,500 per group, the standard error of the mean difference is $\mathrm{SE}\;=\;\sqrt{2/2,500}\;\approx \;0.028$ , indicating that a bias below 0.015 represents a negligible fraction of the typical standard errors in large samples.

All analyses were conducted using R (R Core Team, 2024, Version 4.3.3). The sirt package (Robitzsch, 2025d, Version 4.2-114) was employed to estimate IRT models, including the HAB, HAE, CC, and REG implementations. The package was installed from its GitHub repository. Replication material for the simulation study is available at https://osf.io/q86jz.

### Results

#### No IPD

The results for the estimated mean are shown in Table 1, and the results for the *SD* ${\hat{\sigma }}_{2}$ are shown in Supplementary Table S2 for the condition of no IPD, as a function of the number of items I and sample size N . Without IPD, all methods produced unbiased estimates for ${\hat{\mu }}_{2}$ and ${\hat{\sigma }}_{2}$ . The only exception was the LRT method, which showed slight bias above 0.015 for the mean when I=40 and N=500 . Regarding the RMSE for the mean, CC, FC, and HAE with p=0 were approximately as efficient as the reference method, REG. This was followed by LRT, FIX, HAE with p≤1 , and HAB with p=2 , all of which performed satisfactorily. DD and HAB with p=0 or p=0.25 crossed the 125 threshold for both parameters in conditions with I=20 and N≤1,000 . For DD, this loss of efficiency diminished as N and I increased. For HAB, efficiency improved as p and N increased, as expected under the absence of IPD. In contrast, HAE showed little change across p for the mean and remained close to the reference method, REG. For *SD*, HAE was less efficient, although it still performed satisfactorily and improved as p , N , and I increased. The methods CC, FC, LRT, and FIX stayed close to the reference method REG for *SD*.

**Table 1.** Simulation Study: Bias and Relative RMSE for the Estimated Mean ${\hat{\mu }}_{2}$ in the Condition of No Item Parameter Drift (IPD) as a Function of the Number of Items I , and Sample Size N .

Bias																	
						RMSD		HAB, p =					HAE, p =				
I	N	CC	FC	REG	LRT	FIX	DD	0	0.25	0.5	1	2	0	0.25	0.5	1	2
20	500	0.00	−0.01	0.00	0.01	0.00	−0.01	−0.01	−0.01	−0.01	−0.01	−0.01	−0.01	−0.01	−0.01	−0.01	−0.01
	1,000	0.00	−0.01	0.00	0.00	0.00	0.00	−0.01	−0.01	−0.01	−0.01	−0.01	−0.01	−0.01	−0.01	−0.01	−0.01
	2,500	0.00	0.00	0.00	0.00	0.00	0.00	0.00	0.00	0.00	0.00	0.00	0.00	0.00	0.00	0.00	0.00
40	500	0.00	−0.01	0.00	**0.02**	0.00	0.00	−0.01	−0.01	−0.01	−0.01	−0.01	0.00	0.00	0.00	0.00	0.00
	1,000	0.00	−0.01	0.00	0.00	0.00	−0.01	−0.01	−0.01	−0.01	−0.01	−0.01	−0.01	−0.01	−0.01	−0.01	−0.01
	2,500	0.00	0.00	0.00	0.00	0.00	0.00	0.00	0.00	0.00	0.00	0.00	0.00	0.00	0.00	0.00	0.00
Relative RMSE																	
						RMSD		HAB, p =					HAE, p =				
I	N	CC	FC	REG	LRT	FIX	DD	0	0.25	0.5	1	2	0	0.25	0.5	1	2
20	500	100	100	100^‡^	110	103	**133**	**148**	**129**	120	106	104	103	104	103	102	101
	1,000	100	101	100^‡^	110	103	**129**	**134**	**125**	117	106	103	102	102	102	101	101
	2,500	100	100	100^‡^	109	102	124	111	113	109	104	104	100	101	100	100	100
40	500	100	100	100^‡^	107	101	116	**128**	116	110	104	103	102	103	102	101	100
	1,000	100	101	100^‡^	106	101	111	115	111	107	103	102	101	102	102	101	100
	2,500	100	100	100^‡^	104	102	114	105	106	104	102	101	100	101	101	100	100

*Note.* CC = concurrent calibration; FC = fixed calibration; REG = regularized linking using smooth Bayesian information criterion with ε = 0.001; LRT = likelihood-ratio test with Bonferroni correction with significance level 0.05, applied to Haberman linking based on item difficulties with loss function power p = 2; RMSD = root mean square deviation with fixed (FIX) and data-driven (DD) cutoffs. FIX with cutoff value 0.5 (iterative approach) applied to Haebara linking with normal-density weighting ( σ = 1) with loss function power p = 2; DD with cutoff value 1.7 (iterative approach) applied to Haebara linking with normal-density weighting ( σ = 0.5) with loss function power p = 2; HAB = Haberman linking based on item intercepts with loss function power p ; HAE = Haebara linking with normal-density weighting ( σ = 0.5) and loss function power p . RMSE is calculated with REG^‡^ as the reference method. Absolute bias values ≥0.015 and RMSE values ≥125 % are printed in bold.

#### Balanced IPD

Table 2 presents the results for the estimated mean ${\hat{\mu }}_{2}$ under balanced IPD as a function of the number of items I , the percentage of IPD items, the IPD effect size, and the sample size N . For ${\hat{\mu }}_{2}$ , REG, CC, DD, and HAB performed satisfactorily in terms of bias across these conditions. FC, and HAE, with p=2 , exhibited unsatisfactory negative bias primarily at 30% IPD with δ=1.0 . LRT showed unsatisfactory bias, mainly at N=500 . HAE with p=0 was also biased in a single condition ( δ=1.0 , 30% IPD, I=20 , N=500 ). For RMSE, in the 30% IPD, δ=0.5 conditions, CC, FC, FIX, HAB, with p=2 , and HAE, for all p , were more efficient than REG, particularly at smaller N . In the 30% IPD, δ=1.0 conditions, FC, FIX, and HAE with p=2 showed unsatisfactory RMSE at larger N . For HAB, with p≤0.25 , RMSE was generally unsatisfactory at smaller N , but improved as p or N increased. DD was again unsatisfactory in conditions with I=20 .

**Table 2.** Simulation Study: Bias and Relative RMSE for the Estimated Mean ${\hat{\mu }}_{2}$ in the Condition of Balanced Item Parameter Drift (IPD) as a Function of the Number of Items I , Percentage of IPD Items (%IPD), IPD Effect Size δ , and Sample Size N .

Bias																			
								RMSD		HAB, p =					HAE, p =				
δ	%IPD	I	N	CC	FC	REG	LRT	FIX	DD	0	0.25	0.5	1	2	0	0.25	0.5	1	2
0.5	10	20	500	0.01	−0.01	0.01	**0.02**	0.00	0.00	0.01	0.00	0.00	0.00	0.00	0.00	0.00	0.00	0.00	−0.01
			1,000	0.00	−0.01	0.00	0.01	0.00	0.00	0.00	0.00	0.00	0.00	0.00	0.00	0.00	0.00	0.00	0.00
			2,500	0.00	−0.01	0.00	0.00	0.00	0.00	0.00	0.00	0.00	0.00	0.00	0.00	0.00	0.00	0.00	−0.01
		40	500	0.00	−0.01	0.01	0.01	0.00	0.00	0.00	0.00	0.00	0.00	0.00	0.00	0.00	0.00	0.00	−0.01
			1,000	0.00	−0.01	0.00	0.01	0.00	0.00	0.00	0.00	0.00	0.00	0.00	0.00	0.00	0.00	0.00	−0.01
			2,500	0.00	−0.01	0.00	0.00	0.00	0.00	0.00	0.00	0.00	0.00	0.00	0.00	0.00	0.00	0.00	0.00
	30	20	500	0.01	−**0.02**	0.01	**0.03**	0.00	0.01	0.01	0.00	0.00	0.00	0.00	0.00	0.00	0.00	0.00	−0.01
			1,000	0.00	−**0.02**	0.00	0.01	−0.01	0.00	0.00	−0.01	−0.01	−0.01	−0.01	0.00	0.00	0.00	−0.01	−**0.02**
			2,500	0.00	−0.01	0.00	0.00	0.00	0.00	0.00	0.00	0.00	0.00	0.00	0.00	0.00	0.00	0.00	−0.01
		40	500	0.00	−**0.02**	0.00	**0.02**	−0.01	0.00	0.00	−0.01	−0.01	−0.01	−0.01	−0.01	−0.01	−0.01	−0.01	−**0.02**
			1,000	0.00	−**0.02**	0.00	0.01	0.00	0.00	0.00	0.00	0.00	0.00	0.00	0.00	0.00	0.00	−0.01	−**0.02**
			2,500	0.00	−**0.02**	0.00	0.00	0.01	0.00	0.00	0.00	0.00	0.00	0.00	0.00	0.00	0.00	0.00	−**0.02**
Bias																			
								RMSD		HAB, p =					HAE, p =				
δ	%IPD	I	N	CC	FC	REG	LRT	FIX	DD	0	0.25	0.5	1	2	0	0.25	0.5	1	2
1.0	10	20	500	0.01	−**0.03**	0.01	**0.02**	−0.01	0.00	0.00	0.00	0.00	0.00	0.00	0.00	0.00	0.00	0.00	−**0.02**
			1,000	0.00	−**0.02**	0.00	0.01	−0.01	0.00	−0.01	−0.01	−0.01	−0.01	0.00	0.00	0.00	0.00	0.00	−**0.02**
			2,500	0.00	−**0.02**	0.00	0.01	−0.01	0.00	0.00	0.00	0.00	0.00	0.00	0.00	0.00	0.00	0.00	−**0.02**
		40	500	0.01	−**0.02**	0.01	**0.02**	−0.01	0.00	0.00	0.00	0.00	0.00	0.00	0.00	0.00	0.00	0.00	−**0.02**
			1,000	0.00	−**0.02**	0.00	0.00	−0.01	0.00	0.00	−0.01	−0.01	−0.01	−0.01	0.00	0.00	0.00	0.00	−**0.02**
			2,500	0.00	−**0.02**	0.00	0.00	−0.01	0.00	0.00	0.00	0.00	0.00	0.00	0.00	0.00	0.00	0.00	−**0.02**
	30	20	500	0.01	−**0.06**	0.01	**0.02**	−**0.03**	0.00	0.01	0.00	0.00	0.00	0.00	**0.02**	0.01	0.01	0.00	−**0.05**
			1,000	0.00	−**0.06**	0.00	0.01	−**0.04**	0.00	0.00	0.00	0.00	0.00	0.00	0.01	0.01	0.00	0.00	−**0.05**
			2,500	0.00	−**0.05**	0.00	0.00	−**0.04**	0.00	0.00	0.00	0.00	0.00	0.00	0.01	0.01	0.00	0.00	−**0.05**
		40	500	0.00	−**0.06**	0.00	**0.02**	−**0.03**	−0.01	−0.01	−0.01	−0.01	−0.01	−0.01	0.01	0.00	0.00	−0.01	−**0.06**
			1,000	0.00	−**0.05**	0.00	0.01	−**0.04**	0.00	0.00	0.00	0.00	0.00	0.00	0.01	0.00	0.00	−0.01	−**0.05**
			2,500	0.00	−**0.05**	0.00	0.00	−**0.04**	0.00	0.00	0.00	0.00	0.00	0.00	0.01	0.00	0.00	0.00	−**0.05**

Relative RMSE																			
								RMSD		HAB, p =					HAE, p =				
δ	%IPD	I	N	CC	FC	REG	LRT	FIX	DD	0	0.25	0.5	1	2	0	0.25	0.5	1	2
0.5	10	20	500	97	97	100^‡^	113	98	**133**	**153**	**134**	123	108	102	101	103	101	99	97
			1,000	99	99	100^‡^	110	101	**131**	**128**	122	114	105	103	101	102	101	100	100
			2,500	99	100	100^‡^	108	100	**132**	114	116	111	106	104	101	102	101	101	100
		40	500	100	99	100^‡^	107	102	115	**132**	119	112	104	102	100	101	100	99	100
			1,000	99	100	100^‡^	108	101	114	119	115	109	102	100	100	100	100	99	100
			2,500	100	101	100^‡^	106	102	117	107	109	106	103	102	101	102	101	101	101
	30	20	500	93	93	100^‡^	115	98	**138**	**152**	**129**	118	101	94	98	99	97	94	93
			1,00	94	96	100^‡^	114	101	**127**	**134**	**127**	116	104	98	98	100	99	96	96
			2,500	97	102	100^‡^	114	121	**134**	117	118	113	106	100	103	104	103	101	102
		40	500	96	97	100^‡^	108	98	117	**139**	120	113	102	98	97	99	98	96	97
			1,000	96	99	100^‡^	109	100	117	120	115	110	103	98	98	99	99	98	99
			2,500	99	106	100^‡^	107	**129**	122	109	111	108	104	101	101	102	102	101	106
Relative RMSE																			
								RMSD		HAB, p =					HAE, p =				
δ	%IPD	I	N	CC	FC	REG	LRT	FIX	DD	0	0.25	0.5	1	2	0	0.25	0.5	1	2
1.0	10	20	500	101	102	100^‡^	113	102	**130**	**150**	**131**	121	106	103	104	106	104	102	102
			1,000	100	104	100^‡^	111	102	**135**	**131**	123	116	107	106	102	104	103	101	103
			2,500	100	106	100^‡^	110	104	**134**	113	115	110	105	103	102	102	101	101	106
		40	500	101	101	100^‡^	108	102	116	**134**	119	113	104	102	102	102	101	100	101
			1,000	101	106	100^‡^	106	104	115	118	116	110	104	103	102	103	102	102	106
			2,500	100	110	100^‡^	106	106	115	108	110	107	103	102	101	102	101	101	109
	30	20	500	102	111	100^‡^	120	104	**132**	**152**	**134**	124	109	105	109	109	106	102	109
			1,000	100	**126**	100^‡^	114	112	**139**	**145**	**134**	**126**	113	106	105	105	104	101	124
			2,500	99	**151**	100^‡^	114	**130**	**138**	118	120	114	107	101	107	104	103	101	**149**
		40	500	102	118	100^‡^	111	107	119	**138**	123	115	105	102	105	105	104	102	117
			1,000	102	**130**	100^‡^	108	116	121	121	117	111	105	103	103	103	102	100	**129**
			2,500	102	**167**	100^‡^	106	**144**	114	109	111	108	104	103	105	102	102	101	**169**

*Note.* CC = concurrent calibration; FC = fixed calibration; REG = regularized linking using smooth Bayesian information criterion with ε = 0.001; LRT = likelihood-ratio test with Bonferroni correction with significance level 0.05, applied to Haberman linking based on item difficulties with loss function power p = 2; RMSD = root mean square deviation with fixed (FIX) and data-driven (DD) cutoffs. FIX with cutoff value 0.5 (iterative approach) applied to Haebara linking with normal-density weighting ( σ = 1) with loss function power p = 2; DD with cutoff value 1.7 (iterative approach) applied to Haebara linking with normal-density weighting ( σ = 0.5) with loss function power p = 2; HAB = Haberman linking based on item intercepts with loss function power p ; HAE = Haebara linking with normal-density weighting ( σ = 0.5) and loss function power p . RMSE is calculated with REG^‡^ as the reference method. Values with a gray background indicate an RMSE below 98.5%. Absolute bias values ≥0.015 and RMSE values ≥125 % are printed in bold.

Table 3 presents the results for the estimated *SD*, ${\hat{\sigma }}_{2}$ , under balanced IPD. REG, LRT, DD, and HAB, for all p , remained unbiased, as did HAE, with p≤1 . CC, FC, and FIX showed unsatisfactory negative bias and elevated RMSE when 30% of items drifted or when δ=1.0 . For RMSE, LRT, HAB, and HAE, with p≥1 , performed satisfactorily across all conditions, whereas DD remained the most inefficient, with the highest RMSE values. HAB, with p≤.5 , was unsatisfactory at N≤1,000 , and HAE with p≤.5 was unsatisfactory at I=20 and 30% IPD.

**Table 3.** Simulation Study: Bias and Relative RMSE for the Estimated Standard Deviation ${\hat{\sigma }}_{2}$ in the Condition of Balanced Item Parameter Drift (IPD) as a Function of the Number of Items I , Percentage of IPD Items (%IPD), IPD Effect Size δ , and Sample Size N .

Bias																			
								RMSD		HAB, p =					HAE, p =				
δ	%IPD	I	N	CC	FC	REG	LRT	FIX	DD	0	0.25	0.5	1	2	0	0.25	0.5	1	2
0.5	10	20	500	0.00	−0.01	0.01	0.01	0.01	0.01	0.01	0.01	0.01	0.01	0.01	0.00	0.00	0.00	0.00	0.00
			1,000	0.00	−0.01	0.00	0.00	0.00	0.01	0.01	0.00	0.00	0.00	0.00	0.00	0.00	0.00	0.00	0.00
			2,500	−0.01	−0.01	0.00	0.00	0.00	0.00	0.00	0.00	0.00	0.00	0.00	0.00	0.00	0.00	0.00	0.00
		40	500	0.00	−0.01	0.00	0.01	0.00	0.00	0.01	0.01	0.01	0.01	0.01	0.00	0.00	0.00	0.00	0.00
			1,000	0.00	−0.01	0.00	0.00	0.00	0.00	0.00	0.00	0.00	0.00	0.00	0.00	0.00	0.00	0.00	0.00
			2,500	0.00	0.00	0.00	0.00	0.00	0.00	0.00	0.00	0.00	0.00	0.00	0.00	0.00	0.00	0.00	0.00
	30	20	500	−0.01	−**0.02**	0.00	0.01	0.00	0.01	0.01	0.01	0.01	0.01	0.01	0.00	0.00	0.00	0.00	0.00
			1,000	−0.01	−0.01	0.00	0.00	0.00	0.01	0.00	0.00	0.00	0.00	0.00	0.00	0.00	0.00	0.00	0.00
			2,500	−**0.02**	−0.01	0.00	0.00	−**0.02**	0.00	0.00	0.00	0.00	0.00	0.00	−0.01	0.00	0.00	0.00	0.00
		40	500	−0.01	−**0.02**	0.00	0.00	−0.01	−0.01	0.00	0.00	0.00	0.00	0.00	−0.01	0.00	0.00	0.00	0.00
			1,000	−0.01	−**0.02**	0.00	0.00	−0.01	0.00	0.00	0.00	0.00	0.00	0.00	−0.01	−0.01	−0.01	−0.01	0.00
			2,500	−0.01	−0.01	0.00	0.00	−**0.02**	0.00	0.00	0.00	0.00	0.00	0.00	−0.01	0.00	0.00	0.00	0.00
1.0	10	20	500	−**0.02**	−**0.03**	0.00	0.01	0.00	0.00	0.01	0.01	0.01	0.01	0.01	0.00	0.00	0.00	0.00	0.00
			1,000	−**0.02**	−**0.02**	0.00	0.00	0.00	0.01	0.00	0.00	0.00	0.00	0.00	0.00	0.00	0.00	0.00	0.00
			2,500	−**0.02**	−**0.02**	0.00	0.00	−0.01	0.00	0.00	0.00	0.00	0.00	0.00	0.00	0.00	0.00	0.00	0.00
		40	500	−0.01	−**0.02**	0.01	0.01	0.00	0.01	0.01	0.01	0.01	0.01	0.01	0.01	0.01	0.01	0.00	0.00
			1,000	−**0.02**	−**0.02**	0.00	0.00	−0.01	0.00	0.00	0.00	0.00	0.00	0.00	0.00	0.00	0.00	0.00	0.00
			2,500	−**0.02**	−0.01	0.00	0.00	−0.01	0.00	0.00	0.00	0.00	0.00	0.00	0.00	0.00	0.00	0.00	0.00
	30	20	500	−**0.06**	−**0.05**	0.00	0.01	−**0.02**	0.00	0.01	0.01	0.01	0.00	0.01	0.00	0.00	0.00	−0.01	0.00
			1,000	−**0.06**	−**0.05**	0.00	0.00	−**0.02**	0.00	0.01	0.00	0.00	0.00	0.00	0.00	0.00	0.00	0.00	0.00
			2,500	−**0.06**	−**0.04**	0.00	0.00	−**0.03**	0.00	0.00	0.00	0.00	0.00	0.00	0.00	0.00	0.00	0.00	0.00
		40	500	−**0.05**	−**0.05**	0.00	0.00	−**0.02**	0.00	0.00	0.00	0.00	0.00	0.00	0.00	0.00	0.00	−0.01	0.00
			1,000	−**0.05**	−**0.04**	0.00	0.00	−**0.03**	0.00	0.00	0.00	0.00	0.00	0.00	0.00	0.00	0.00	−0.01	0.00
			2,500	−**0.05**	−**0.04**	0.00	0.00	−**0.03**	0.00	0.00	0.00	0.00	0.00	0.00	0.00	0.00	0.00	0.00	0.00
Relative RMSE																			
								RMSD		HAB, p =					HAE, p =				
δ	%IPD	I	N	CC	FC	REG	LRT	FIX	DD	0	0.25	0.5	1	2	0	0.25	0.5	1	2
0.5	10	20	500	99	100	100^‡^	104	104	**167**	**164**	**138**	127	110	105	117	121	116	111	110
			1,000	99	101	100^‡^	105	103	**167**	**141**	**128**	119	107	102	114	117	115	112	111
			2,500	101	102	100^‡^	106	103	**168**	117	120	113	105	102	114	115	114	112	111
		40	500	99	102	100^‡^	106	105	**135**	**143**	**125**	116	105	103	109	111	109	107	106
			1,000	100	103	100^‡^	104	102	**138**	124	119	113	105	102	110	112	111	109	108
			2,500	100	103	100^‡^	104	103	**145**	107	110	107	103	102	111	111	111	110	109
	30	20	500	97	100	100^‡^	108	104	**184**	**161**	**137**	**127**	110	103	**128**	**131**	125	116	109
			1,000	100	102	100^‡^	113	108	**182**	**135**	**126**	116	104	102	124	**126**	123	116	111
			2,500	109	108	100^‡^	112	**130**	**182**	118	120	114	106	102	**126**	**126**	124	120	114
		40	500	99	103	100^‡^	105	103	**139**	**143**	**126**	117	105	103	119	121	117	111	106
			1,000	102	105	100^‡^	104	107	**147**	120	114	109	103	101	115	116	114	110	107
			2,500	107	106	100^‡^	107	**141**	**153**	107	109	106	102	101	118	116	115	112	107
1.0	10		500	100	104	100^‡^	107	103	**163**	**158**	**136**	**125**	108	104	119	122	118	114	112
		20	1,000	102	104	100^‡^	106	102	**173**	**140**	**129**	119	106	103	116	119	117	114	111
			2,500	113	112	100^‡^	106	106	**168**	117	119	113	105	102	114	114	114	114	114
			500	99	102	100^‡^	106	104	**138**	**146**	**129**	119	106	104	111	114	111	107	105
		40	1,000	104	107	100^‡^	103	104	**138**	119	115	109	102	101	112	113	112	111	109
			2,500	112	110	100^‡^	104	103	**141**	107	110	106	103	102	109	110	109	108	107
	30	20	500	120	119	100^‡^	118	108	**187**	**164**	**139**	**128**	111	104	**136**	**139**	**132**	123	112
			1,000	**140**	**128**	100^‡^	113	109	**187**	**145**	**129**	120	107	103	**126**	**129**	**125**	120	110
			2,500	**192**	**157**	100^‡^	112	124	**188**	116	118	111	104	102	124	**125**	124	121	111
		40	500	119	119	100^‡^	108	106	**149**	**141**	124	115	105	102	119	121	118	113	106
			1,000	**140**	**130**	100^‡^	109	111	**152**	123	118	111	104	102	117	118	116	113	104
			2,500	**199**	**167**	100^‡^	110	**134**	**143**	106	109	105	102	101	115	116	115	114	107

*Note.* CC = concurrent calibration; FC = fixed calibration; REG = regularized linking using smooth Bayesian information criterion with ε = 0.001; LRT = likelihood-ratio test with Bonferroni correction with significance level 0.05, applied to Haberman linking based on item difficulties with loss function power p = 2; RMSD = root mean square deviation with fixed (FIX) and data-driven (DD) cutoffs. FIX with cutoff value 0.5 (iterative approach) applied to Haebara linking with normal-density weighting ( σ = 1) with loss function power p = 2; DD with cutoff value 1.7 (iterative approach) applied to Haebara linking with normal-density weighting ( σ = 0.5) with loss function power p = 2; HAB = Haberman linking based on item intercepts with loss function power p ; HAE = Haebara linking with normal-density weighting ( σ = 0.5) and loss function power p . RMSE is calculated with REG^‡^ as the reference method. Values with a gray background indicate an RMSE below 98.5%. Absolute bias values ≥0.015 and RMSE values ≥125 % are printed in bold.

#### Unbalanced IPD

Table 4 displays the results for the estimated mean ${\hat{\mu }}_{2}$ under unbalanced IPD as a function of the number of items I , the percentage of IPD items, the IPD effect size, and sample size N . No method was uniformly satisfactory for the mean ${\hat{\mu }}_{2}$ . Regarding bias, HAB with p=0 performed best overall, with only three unsatisfactory values up to 0.05 at N=500 and 1,000 , when 30% of the items drifted and δ=0.5 . DD and REG showed satisfactory bias at N=2,500 , with only a few exceptions at N≤1,000 . The bias of CC, FC, and LRT increased with %IPD and δ , reaching values as high as 0.28, 0.21, and 0.18, respectively. For LRT, however, the bias decreased as the sample size increased. For both HAB and HAE, the bias increased as the loss function power p increased. Consequently, specifications with p=2 were the most biased, with values up to 0.31 for HAB and up to 0.15 for HAE. In terms of RMSE, most methods performed unsatisfactorily whenever 30% of items drifted and/or δ=1.0 held. Methods that exhibited the largest biases under the 30% IPD, δ=1.0 condition—CC, FC, LRT, and HAB/HAE with p≥1 —also exhibited high RMSE, often surpassing 300 at N=2,500 . Satisfactory performance relative to REG was largely confined to the 10% IPD, δ=0.5 case, where FC, FIX, and most HAE with p≤1 were adequate. For HAB, this occurred only for p=0.5 and 1 .

**Table 4.** Simulation Study: Bias and Relative RMSE for the Estimated Mean ${\hat{\mu }}_{2}$ in the Condition of Unbalanced Item Parameter Drift (IPD) as a Function of the Number of Items I , Percentage of IPD Items (%IPD), IPD Effect Size δ , and Sample Size N .

Bias																			
								RMSD		HAB, p =					HAE, p =				
δ	%IPD	I	N	CC	FC	REG	LRT	FIX	DD	0	0.25	0.5	1	2	0	0.25	0.5	1	2
0.5	10	20	500	**0.05**	**0.03**	**0.02**	**0.05**	**0.04**	0.01	0.01	**0.02**	**0.02**	**0.03**	**0.05**	**0.02**	**0.02**	**0.02**	**0.02**	**0.03**
			1,000	**0.05**	**0.03**	0.01	**0.02**	**0.03**	0.00	0.00	0.00	0.01	**0.02**	**0.05**	**0.02**	0.01	**0.02**	**0.02**	**0.03**
			2,500	**0.05**	**0.04**	0.00	0.00	**0.03**	0.00	0.00	0.00	0.00	0.01	**0.05**	0.01	0.01	0.01	**0.02**	**0.03**
		40	500	**0.05**	**0.03**	0.01	**0.05**	**0.03**	0.00	0.00	0.01	0.01	**0.02**	**0.04**	**0.02**	**0.02**	**0.02**	**0.02**	**0.03**
			1,000	**0.05**	**0.04**	0.01	**0.03**	**0.03**	0.00	0.00	0.01	0.01	**0.02**	**0.05**	**0.02**	**0.02**	**0.02**	**0.02**	**0.03**
			2,500	**0.05**	**0.04**	0.00	0.00	**0.03**	0.00	0.00	0.00	0.00	0.01	**0.05**	**0.02**	0.01	0.01	**0.02**	**0.03**
	30	20	500	**0.14**	**0.11**	**0.05**	**0.15**	**0.09**	**0.06**	**0.05**	**0.07**	**0.08**	**0.11**	**0.15**	**0.08**	**0.08**	**0.08**	**0.08**	**0.09**
			1,000	**0.14**	**0.12**	**0.02**	**0.12**	**0.10**	**0.03**	**0.02**	**0.04**	**0.06**	**0.10**	**0.15**	**0.07**	**0.06**	**0.07**	**0.08**	**0.09**
			2,500	**0.14**	**0.12**	0.00	**0.04**	**0.10**	0.01	0.00	**0.02**	**0.03**	**0.07**	**0.15**	**0.06**	**0.05**	**0.05**	**0.07**	**0.09**
		40	500	**0.14**	**0.11**	**0.05**	**0.14**	**0.08**	**0.05**	**0.04**	**0.07**	**0.08**	**0.11**	**0.15**	**0.08**	**0.08**	**0.08**	**0.08**	**0.09**
			1,000	**0.15**	**0.12**	**0.02**	**0.13**	**0.10**	0.01	0.01	**0.04**	**0.05**	**0.09**	**0.15**	**0.07**	**0.06**	**0.07**	**0.08**	**0.09**
			2,500	**0.14**	**0.12**	0.00	**0.05**	**0.10**	0.00	0.00	**0.02**	**0.03**	**0.07**	**0.15**	**0.06**	**0.05**	**0.05**	**0.07**	**0.09**
1.0	10	20	500	**0.09**	**0.06**	0.01	**0.02**	**0.06**	0.00	0.00	0.00	0.01	**0.02**	**0.10**	**0.02**	**0.02**	**0.02**	**0.03**	**0.05**
			1,000	**0.09**	**0.07**	0.00	0.01	**0.06**	0.00	0.00	0.00	0.00	**0.02**	**0.10**	0.01	0.01	**0.02**	**0.02**	**0.05**
			2500	**0.09**	**0.07**	0.00	0.01	**0.06**	0.00	0.00	0.00	0.00	0.01	**0.10**	0.01	0.01	0.01	**0.02**	**0.05**
		40	500	**0.09**	**0.06**	0.01	**0.02**	**0.04**	−0.01	0.00	0.01	0.01	**0.03**	**0.10**	**0.02**	**0.02**	**0.02**	**0.03**	**0.05**
			1,000	**0.09**	**0.06**	0.00	0.01	**0.06**	−0.01	−0.01	0.00	0.00	**0.02**	**0.10**	0.01	0.01	0.01	**0.02**	**0.05**
			2,500	**0.08**	**0.07**	0.00	0.00	**0.06**	−0.01	0.00	0.00	0.00	0.01	**0.10**	0.01	0.01	0.01	**0.02**	**0.05**
	30	20	500	**0.28**	**0.20**	0.01	**0.17**	**0.04**	**0.02**	0.00	**0.03**	**0.05**	**0.12**	**0.30**	**0.07**	**0.07**	**0.08**	**0.10**	**0.15**
			1,000	**0.28**	**0.20**	0.00	**0.07**	0.01	0.00	0.00	**0.02**	**0.03**	**0.09**	**0.30**	**0.06**	**0.05**	**0.06**	**0.09**	**0.15**
			2,500	**0.27**	**0.21**	0.00	**0.07**	0.00	0.01	0.00	0.01	**0.02**	**0.07**	**0.31**	**0.04**	**0.03**	**0.04**	**0.07**	**0.15**
		40	500	**0.28**	**0.20**	0.00	**0.18**	**0.05**	0.00	0.00	**0.03**	**0.05**	**0.12**	**0.30**	**0.07**	**0.07**	**0.08**	**0.10**	**0.15**
			1,000	**0.28**	**0.21**	0.00	**0.07**	0.01	0.00	0.00	0.01	**0.03**	**0.09**	**0.30**	**0.05**	**0.05**	**0.06**	**0.09**	**0.15**
			2,500	**0.27**	**0.21**	0.00	**0.07**	0.00	0.00	0.00	0.01	**0.02**	**0.06**	**0.31**	**0.04**	**0.03**	**0.04**	**0.07**	**0.15**
Relative RMSE																			
								RMSD		HAB, p =					HAE, p =				
δ	%IPD	I	N	CC	FC	REG	LRT	FIX	DD	0	0.25	0.5	1	2	0	0.25	0.5	1	2
0.5	10	20	500	112	102	100^‡^	114	106	**130**	**151**	**134**	123	110	112	103	104	103	101	102
			1,000	121	110	100^‡^	116	111	**139**	**133**	**125**	117	109	123	104	105	104	103	106
			2,500	**150**	**134**	100^‡^	111	**130**	**137**	116	119	114	112	**160**	108	106	106	108	122
		40	500	113	103	100^‡^	**125**	105	113	**130**	117	111	106	112	102	103	102	101	101
			1,000	**131**	117	100^‡^	115	116	111	114	111	108	108	**132**	105	105	105	106	111
			2,500	**158**	**141**	100^‡^	105	**133**	116	107	108	106	109	**167**	109	106	107	110	**127**
	30	20	500	**146**	118	100^‡^	**154**	114	**138**	**154**	**139**	**134**	**133**	**150**	106	107	106	104	105
			1,000	**204**	**170**	100^‡^	**185**	**157**	**155**	**133**	**135**	**135**	**157**	**215**	**128**	123	**125**	**131**	**144**
			2,500	**340**	**287**	100^‡^	**168**	**253**	**165**	116	**128**	**131**	**188**	**369**	**176**	**149**	**158**	**182**	**232**
		40	500	**152**	125	100^‡^	**155**	111	115	**136**	**131**	**127**	**134**	**155**	109	109	108	108	110
			1,000	**221**	**185**	100^‡^	**200**	**162**	121	112	124	**128**	**162**	**233**	**134**	**127**	**130**	**138**	**154**
			2,500	**379**	**322**	100^‡^	**179**	**278**	120	106	117	**126**	**200**	**411**	**191**	**156**	**168**	**198**	**256**
1.0	10	20	500	**140**	117	100^‡^	114	120	**136**	**153**	**136**	**126**	113	**147**	105	106	105	104	111
			1,000	**168**	**141**	100^‡^	111	**138**	**139**	**132**	**125**	117	111	**182**	105	106	106	108	**127**
			2,500	**231**	**191**	100^‡^	112	**176**	**135**	112	115	111	112	**264**	106	104	106	111	**161**
		40	500	**145**	122	100^‡^	109	118	114	**133**	121	114	109	**152**	104	105	104	105	114
			1,000	**174**	**144**	100^‡^	107	**137**	117	121	116	111	108	**191**	104	104	105	107	**127**
			2,500	**236**	**195**	100^‡^	105	**176**	117	108	111	107	109	**270**	104	103	104	109	**160**
	30	20	500	**314**	**229**	100^‡^	**224**	125	**198**	**157**	**150**	**146**	**180**	**335**	**136**	**137**	**140**	**151**	**184**
			1,000	**426**	**318**	100^‡^	**169**	108	**194**	**146**	**141**	**136**	**181**	**465**	**135**	**133**	**141**	**167**	**246**
			2,500	**650**	**493**	100^‡^	**236**	103	**155**	119	124	124	**193**	**724**	**148**	**133**	**147**	**200**	**370**
		40	500	**333**	**247**	100^‡^	**241**	122	**160**	**144**	**134**	**135**	**179**	**357**	**132**	**133**	**138**	**154**	**193**
			1,000	**445**	**339**	100^‡^	**162**	104	**152**	123	122	123	**181**	**486**	**136**	**132**	**141**	**172**	**257**
			2,500	**687**	**530**	100^‡^	**222**	102	**129**	108	112	115	**192**	**764**	**150**	**133**	**149**	**207**	**391**

*Note.* CC = concurrent calibration; FC = fixed calibration; REG = regularized linking using smooth Bayesian information criterion with ε = 0.001; LRT = likelihood-ratio test with Bonferroni correction with significance level 0.05, applied to Haberman linking based on item difficulties with loss function power p = 2; RMSD = root mean square deviation with fixed (FIX) and data-driven (DD) cutoffs. FIX with cutoff value 0.5 (iterative approach) applied to Haebara linking with normal-density weighting ( σ = 1) with loss function power p = 2; DD with cutoff value 1.7 (iterative approach) applied to Haebara linking with normal-density weighting ( σ = 0.5) with loss function power p = 2; HAB = Haberman linking based on item intercepts with loss function power p ; HAE = Haebara linking with normal-density weighting ( σ = 0.5) and loss function power p . RMSE is calculated with REG^‡^ as the reference method. Absolute bias values ≥0.015 and RMSE values ≥125 % are printed in bold.

The results for the estimated *SD* ${\hat{\sigma }}_{2}$ are shown in Table 5 for unbalanced IPD. REG and HAB (across all p ) were unbiased. Although HAE performed unsatisfactorily when 30% of the items drifted, it performed its best at p=.25 , followed by 0.5 . FC and CC displayed unsatisfactorily negative bias that worsened with the percentage of IPD items and with δ . FIX bias decreased at 30% IPD and δ=1.0 as N increased. DD was biased in five of the 12 30% IPD cells. For RMSE, HAB with p≥1 performed satisfactorily across all conditions. All other non-reference methods became unsatisfactory for at least some N when 30% of the items drifted and/or when δ=1.0 was applied. FC and HAE exhibited the highest RMSE, particularly at 30% IPD and δ=1.0 with N=2,500 . DD was inefficient throughout, with RMSE regularly above 160. No method outperformed REG.

**Table 5.** Simulation Study: Bias and Relative RMSE for the Estimated Standard Deviation ${\hat{\sigma }}_{2}$ in the Condition of Unbalanced Item Parameter Drift (IPD) as a Function of the Number of Items I , Percentage of IPD Items (%IPD), IPD Effect Size δ , and Sample Size N .

Bias																			
								RMSD		HAB, p =					HAE, p =				
δ	%IPD	I	N	CC	FC	REG	LRT	FIX	DD	0	0.25	0.5	1	2	0	0.25	0.5	1	2
0.5	10	20	500	−0.01	−**0.03**	0.00	0.01	−**0.02**	0.01	0.01	0.01	0.01	0.01	0.01	0.00	0.00	−0.01	−0.01	−**0.03**
			1,000	−**0.02**	−**0.03**	0.00	0.00	−**0.02**	0.00	0.00	0.00	0.00	0.00	0.00	0.00	0.00	0.00	−0.01	−**0.04**
			2,500	−**0.02**	−**0.02**	0.00	0.00	−**0.03**	0.00	0.00	0.00	0.00	0.00	0.00	0.00	0.00	0.00	−0.01	−**0.04**
		40	500	−0.01	−**0.03**	0.00	0.01	−**0.02**	−0.01	0.00	0.00	0.00	0.00	0.01	−0.01	0.00	−0.01	−**0.02**	−**0.04**
			1,000	−0.01	−**0.02**	0.00	0.00	−**0.02**	0.00	0.00	0.00	0.00	0.00	0.00	0.00	0.00	0.00	−0.01	−**0.03**
			2,500	−**0.02**	−**0.02**	0.00	0.00	−**0.03**	0.00	0.00	0.00	0.00	0.00	0.00	0.00	0.00	0.00	−0.01	−**0.04**
	30	20	500	−**0.04**	−**0.07**	−0.01	0.01	−**0.07**	−**0.03**	0.00	0.00	0.00	0.01	0.01	−**0.04**	−**0.03**	−**0.05**	−**0.07**	−**0.10**
			1,000	−**0.04**	−**0.06**	0.00	0.00	−**0.08**	−0.01	0.01	0.01	0.01	0.01	0.01	−**0.04**	−**0.02**	−**0.03**	−**0.06**	−**0.10**
			2,500	−**0.05**	−**0.07**	0.00	−0.01	−**0.09**	−0.01	0.00	0.00	0.00	0.00	0.00	−**0.04**	−**0.02**	−**0.03**	−**0.06**	−**0.11**
		40	500	−**0.04**	−**0.07**	−0.01	0.00	−**0.07**	−**0.06**	0.00	0.00	0.00	0.01	0.01	−**0.04**	−**0.03**	−**0.04**	−**0.07**	−**0.10**
			1,000	−**0.04**	−**0.07**	−0.01	0.00	−**0.09**	−**0.03**	0.00	0.00	0.00	0.00	0.00	−**0.05**	−**0.03**	−**0.04**	−**0.07**	−**0.11**
			2,500	−**0.04**	−**0.06**	0.00	0.00	−**0.09**	0.00	0.00	0.00	0.00	0.00	0.00	−**0.04**	−0.01	−**0.03**	−**0.05**	−**0.10**
1.0	10	20	500	−**0.03**	−**0.05**	0.01	0.01	−**0.04**	0.01	0.01	0.01	0.01	0.01	0.01	**0.02**	0.01	0.01	−0.01	−**0.06**
			1,000	−**0.04**	−**0.05**	0.00	0.00	−**0.06**	0.00	0.00	0.00	0.00	0.00	0.00	0.01	0.01	0.00	−0.01	−**0.06**
			2,500	−**0.04**	−**0.05**	0.00	0.00	−**0.06**	0.00	0.00	0.00	0.00	0.00	0.00	0.01	0.00	0.00	−0.01	−**0.06**
		40	500	−**0.03**	−**0.05**	0.00	0.00	−**0.04**	0.00	0.01	0.01	0.00	0.00	0.00	0.01	0.01	0.00	−0.01	−**0.06**
			1,000	−**0.04**	−**0.05**	0.00	0.00	−**0.06**	0.00	0.00	0.00	0.00	0.00	0.00	0.01	0.00	0.00	−0.01	−**0.07**
			2,500	−**0.04**	−**0.05**	0.00	0.00	−**0.06**	0.00	0.00	0.00	0.00	0.00	0.00	0.00	0.00	0.00	−0.01	−**0.07**
	30	20	500	−**0.09**	−**0.14**	0.01	−0.01	−**0.02**	−0.01	0.01	0.01	0.01	0.01	0.01	**0.03**	0.01	−0.01	−**0.07**	−**0.19**
			1,000	−**0.09**	−**0.14**	0.00	−**0.02**	0.00	−**0.02**	0.00	0.00	0.00	0.00	0.00	**0.02**	0.01	−0.01	−**0.06**	−**0.19**
			2,500	−**0.10**	−**0.13**	0.00	−**0.03**	0.00	−0.01	0.00	0.00	0.00	0.00	0.00	0.01	0.00	−0.01	−**0.05**	−**0.19**
		40	500	−**0.08**	−**0.13**	0.01	0.00	−**0.02**	−**0.02**	0.01	0.01	0.01	0.01	0.01	**0.03**	0.01	−0.01	−**0.07**	−**0.18**
			1,000	−**0.08**	−**0.12**	0.00	−0.01	−0.01	−0.01	0.00	0.00	0.00	0.00	0.00	**0.02**	0.01	−0.01	−**0.06**	−**0.19**
			2,500	−**0.08**	−**0.12**	0.00	−**0.03**	0.00	0.00	0.00	0.00	0.00	0.00	0.00	**0.02**	0.00	−0.01	−**0.05**	−**0.19**
Relative RMSE																			
								RMSD		HAB, p =					HAE, p =				
δ	%IPD	I	N	CC	FC	REG	LRT	FIX	DD	0	0.25	0.5	1	2	0	0.25	0.5	1	2
0.5	10	20	500	100	107	100^‡^	103	109	**175**	**163**	**140**	**127**	110	103	118	120	117	114	119
			1,000	103	111	100^‡^	107	113	**176**	**139**	**129**	119	107	103	116	120	117	115	**126**
			2,500	109	120	100^‡^	106	**130**	**179**	116	119	112	105	102	114	116	115	116	**148**
		40	500	101	108	100^‡^	107	109	**136**	**142**	123	114	104	103	112	114	112	109	116
			1,000	102	110	100^‡^	104	112	**138**	123	117	110	103	102	109	111	110	109	**125**
			2,500	110	122	100^‡^	104	**136**	**146**	107	109	106	102	101	110	110	110	112	**155**
	30	20	500	108	**130**	100^‡^	102	**136**	**183**	**155**	**136**	124	107	103	**133**	**133**	**132**	**138**	**162**
			1,000	116	**143**	100^‡^	104	**166**	**183**	**143**	**129**	119	105	101	**133**	125	**127**	**145**	**194**
			2,500	**163**	**210**	100^‡^	119	**267**	**197**	118	118	112	105	102	**165**	**131**	**142**	**194**	**310**
		40	500	110	**135**	100^‡^	104	**139**	**163**	**140**	123	114	104	101	**127**	123	**127**	**140**	**175**
			1,000	**129**	**163**	100^‡^	102	**204**	**163**	**126**	120	112	104	100	**146**	**126**	**136**	**169**	**238**
			2,500	**157**	**205**	100^‡^	107	**284**	**149**	105	107	104	101	101	**165**	122	**137**	**198**	**331**
1.0	10	20	500	104	115	100^‡^	108	115	**174**	**159**	**136**	**125**	108	104	120	121	116	111	**128**
			1,000	122	**135**	100^‡^	108	**146**	**179**	**147**	**136**	**125**	110	103	117	119	117	114	**159**
			2,500	**147**	**160**	100^‡^	107	**188**	**173**	115	117	111	104	102	116	115	114	114	**204**
		40	500	109	123	100^‡^	103	121	**139**	**143**	123	114	104	101	113	114	111	109	**139**
			1,000	120	**134**	100^‡^	104	**150**	**140**	121	115	110	104	102	111	111	110	109	**164**
			2,500	**159**	**178**	100^‡^	106	**225**	**142**	113	116	111	105	101	112	111	111	114	**243**
	30	20	500	**146**	**195**	100^‡^	109	**129**	**198**	**162**	**140**	**127**	108	103	**140**	**136**	**129**	**145**	**249**
			1,000	**191**	**258**	100^‡^	**126**	115	**208**	**143**	**131**	120	106	103	**132**	**129**	125	**156**	**345**
			2,500	**283**	**385**	100^‡^	**195**	115	**227**	114	116	109	103	101	**133**	**127**	**127**	**189**	**531**
		40	500	**144**	**196**	100^‡^	104	117	**169**	**139**	121	112	103	102	**129**	124	119	**144**	**269**
			1,000	**191**	**263**	100^‡^	112	110	**159**	124	118	111	103	101	**126**	121	118	**162**	**381**
			2,500	**276**	**385**	100^‡^	**159**	108	**161**	107	110	106	102	100	125	116	115	**193**	**582**

*Note.* CC = concurrent calibration; FC = fixed calibration; REG = regularized linking using smooth Bayesian information criterion with ε = 0.001; LRT = likelihood-ratio test with Bonferroni correction with significance level 0.05, applied to Haberman linking based on item difficulties with loss function power p = 2; RMSD = root mean square deviation with fixed (FIX) and data-driven (DD) cutoffs. FIX with cutoff value 0.5 (iterative approach) applied to Haebara linking with normal-density weighting ( σ = 1) with loss function power p = 2; DD with cutoff value 1.7 (iterative approach) applied to Haebara linking with normal-density weighting ( σ = 0.5) with loss function power p = 2; HAB = Haberman linking based on item intercepts with loss function power p ; HAE = Haebara linking with normal-density weighting ( σ = 0.5) and loss function power p . RMSE is calculated with REG^‡^ as the reference method. Absolute bias values ≥0.015 and RMSE values ≥125 % are printed in bold.

In summary, methods that assume full invariance (CC and FC) performed optimally without IPD but severely degraded under unbalanced IPD. Robust linking methods with small p -values and REG maintained stability across all conditions, although with some loss of efficiency in the no-IPD case. The systematic negative bias in ${\hat{\sigma }}_{2}$ for FC aligns with the findings from Robitzsch (2024a), as the fixed parameters cannot accommodate the increased item-response variability induced by IPD. The negative bias, therefore, leads to an underestimation of the population variance. The superior performance of HAB over HAE for *SD* estimation under uniform IPD reflects the previously discussed differences in estimation. HAB’s advantage over HAE for *SD* estimation under uniform IPD stems from its separate estimation of the variance parameter using only discrimination parameters, which remain unaffected by uniform intercept drift. In contrast, HAE’s simultaneous estimation of both trend parameters makes it more vulnerable to bias propagation from drifting intercepts, as detailed by Robitzsch (2025c).

### Additional Results: Variation of Fixed and Data-Driven RMSD Cutoffs

**Figure 1.**
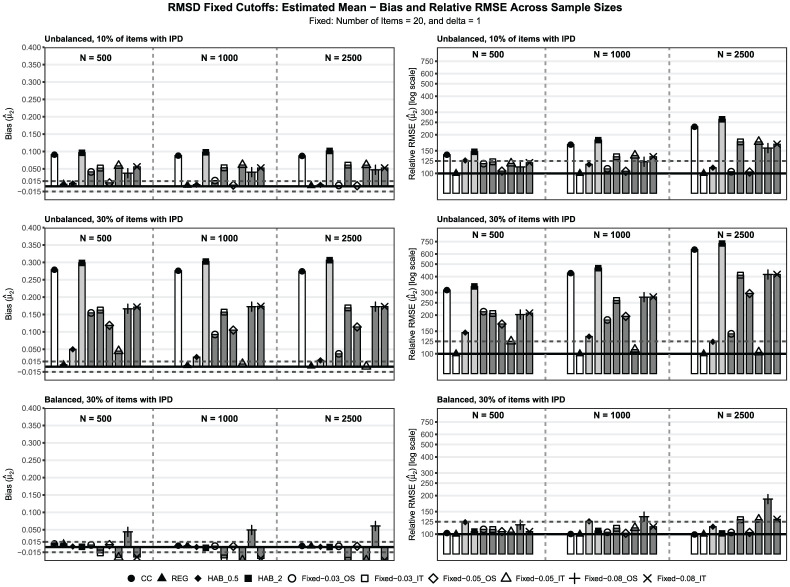
Bias and Relative RMSE for the Estimated Mean ${\hat{\mu }}_{2}$ in the Condition of I = 20 Number of Items, and IPD Effect Size δ = 1 as a Function of IPD Balance (Unbalanced, Balanced), Percentage of IPD Items (%IPD), and Sample Size N . *Note.* CC = concurrent calibration; REG = regularized linking; ${\mathrm{HAB}}_{0.5}$ = Haberman linking based on item intercepts with loss function power p = .5; ${\mathrm{HAB}}_{2}$ = Haberman linking based on item intercepts with loss function power p = 2; Fixed-0.03, Fixed-0.05, Fixed-0.08 = root mean square deviation with fixed cutoff values (0.03, 0.05, 0.08) applied to Haebara linking with normal-density weighting ( σ = 1) and loss function power p = 2, where subscript OS denotes the one-step approach and IT denotes the iterative approach.

**Figure 2.**
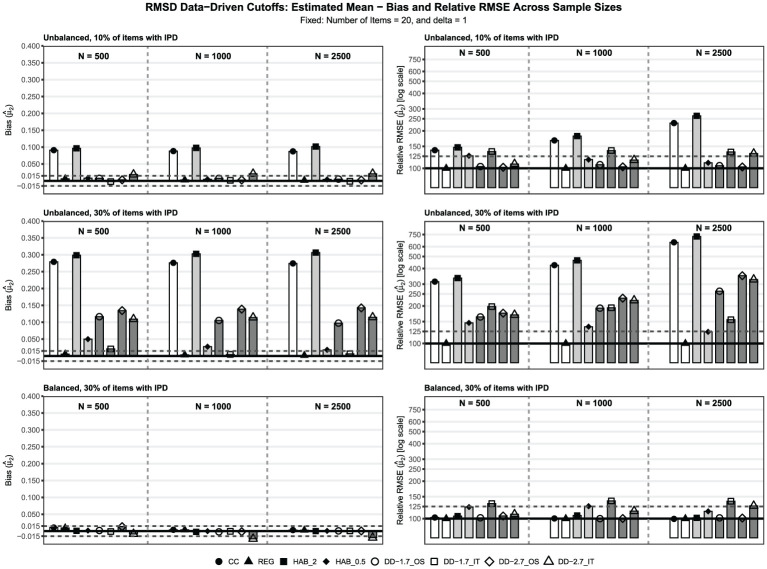
Bias and Relative RMSE for the Estimated Mean ${\hat{\mu }}_{2}$ in the Condition of I = 20 Number of Items, and IPD Effect Size δ = 1 as a Function of IPD Balance (Unbalanced, Balanced), Percentage of IPD Items (%IPD), and Sample Size N . *Note.* CC = concurrent calibration; REG = regularized linking; ${\mathrm{HAB}}_{0.5}$ = Haberman linking based on item intercepts with loss function power p = 0.5; ${\mathrm{HAB}}_{2}$ = Haberman linking based on item intercepts with loss function power p = 2; DD-1.7, DD-2.7 = root mean square deviation with data-driven cutoff values ( τ = 1.7, 2.7) applied to Haebara linking with normal-density weighting ( σ = 0.5) and loss function power p = 2, where subscript OS denotes the one-step approach and IT denotes the iterative approach.

We extend the previous cutoff analysis (FIX 0.05 , DD τ=1.7 ) to include FIX cutoffs 0.03 and 0.08 and DD cutoff τ=2.7 . Each cutoff uses OS and IT approaches, yielding six combinations. The re-estimation procedure under partial invariance remains constant. After fixed RMSD, trends are re-estimated using HAE with normal-density weights ( σ=1 ). After DD RMSD, trends are re-estimated using HAE with normal-density weights ( σ=0.5 ). We compare these results to those of CC, REG, and HAB, based on item intercepts with loss function powers p=0.5 and p=2 . Performance is assessed by bias and RMSE for the trend estimates of the mean ${\hat{\mu }}_{2}$ and the *SD* ${\hat{\sigma }}_{2}$ at T2. Figures 1 and 2 display the bar plots for the estimated mean ( ${\hat{\mu }}_{2}$ ) for FIX and DD RMSD cutoffs, respectively, under both OS and IT, showing three key conditions: unbalanced IPD with 10% and 30% of items exhibiting drift; and balanced IPD with 30% drift; all three with δ=1.0 and I=20 items. Complete tabular results for both ${\hat{\mu }}_{2}$ and ${\hat{\sigma }}_{2}$ under all conditions are provided in the Supplement (Tables S3 to S14).

#### Fixed RMSD Cutoffs

Without IPD, all six combinations yielded satisfactory bias for ${\hat{\mu }}_{2}$ and ${\hat{\sigma }}_{2}$ across sample sizes and item counts. All specifications showed satisfactory RMSE, although 0.03 was slightly elevated at N=500 versus 0.05 and 0.08. The 0.05 and 0.08 cutoffs were similar to the comparison methods under no IPD.

Under balanced IPD, the bias patterns differed between the two trend parameters. For ${\hat{\mu }}_{2}$ , the 0.03 and 0.05 cutoffs with the OS approach maintained satisfactory bias across all conditions. However, the 0.03 IT approach, the 0.05 IT approach, and both 0.08 approaches produced unsatisfactory bias (exceeding 0.015) when 30% of the items exhibited IPD with δ=1.0 . With 0.08, a sign reversal appeared between the OS approach (positive bias) and the IT approach (negative bias) under these conditions. For ${\hat{\sigma }}_{2}$ , no specification achieved satisfactory performance across all conditions. The 0.03 OS approach exhibited unsatisfactory bias of −0.03 in two conditions, while the IT approach showed this in four conditions with 30% IPD and δ=1.0 . The 0.08 cutoffs produced the largest negative bias values, with the IT approach ranging from −0.03 to −0.07 . RMSE became unsatisfactory primarily when bias was elevated, with the 0.08 OS approach performing worst (up to 231). In comparison with the reference methods, for the mean, under the OS approach, with 0.03 and 0.05, the FIX-cutoff estimators were broadly comparable to REG, HAB, and CC; in terms of RMSE, they were weaker than REG and CC, and for the *SD*, they performed similarly to CC, but below REG and HAB, especially HAB, with p=2 .

Under unbalanced IPD, no FIX-cutoff specification provided uniformly satisfactory performance. For ${\hat{\mu }}_{2}$ , positive bias increased with both the percentage of IPD items and the effect size δ across all specifications. The 0.05 IT approach uniquely achieved satisfactory bias when 10% of items drifted with δ=1.0 in larger samples. The 0.03 OS approach occasionally achieved satisfactory bias at N=2,500 , in selected conditions with δ=0.5 . RMSE exceeded the 125 threshold for most specifications when 30% of items drifted, or when δ=1.0 . For ${\hat{\sigma }}_{2}$ , all specifications produced unsatisfactory negative bias. The 0.08 approaches showed the most severe bias, reaching −0.17 with 30% IPD and δ=1.0 . The 0.05 IT approach showed the least bias among FIX cutoffs, but remained unsatisfactory in most conditions with 30% IPD. Under unbalanced IPD, FIX cutoffs generally underperformed relative to REG, HAB, and CC in both bias and RMSE for ${\hat{\mu }}_{2}$ and ${\hat{\sigma }}_{2}$ , with comparability observed only in isolated cases, such as 0.05 with IT for the mean at 10% IPD and δ=1.0 , in larger samples.

Overall, the FIX-cutoff approach performed adequately only under no IPD or under limited, balanced IPD conditions. Among the specifications, 0.05 with OS provided the most satisfactory performance across balanced IPD. IT performed better in specific unbalanced IPD scenarios. The 0.08 cutoff was too lenient under balanced IPD with δ=1.0 and 30% IPD, in both OS and IT.

#### Data-Driven RMSD Cutoffs

Without IPD, all DD specifications maintained satisfactory bias for both trend parameters. RMSE performance varied by cutoff and approach. The τ=2.7 specifications were satisfactory across all conditions. The τ=1.7 IT specification showed unsatisfactory RMSE for ${\hat{\mu }}_{2}$ with I=20 items (reaching 133 at N=500 ) and for ${\hat{\sigma }}_{2}$ in several conditions (up to 169). The OS approach was more efficient than the IT approach. Compared to the reference methods, the bias for ${\hat{\mu }}_{2}$ and ${\hat{\sigma }}_{2}$ was similar. However, for ${\hat{\sigma }}_{2}$ , the RMSE of all RMSD DD specifications was worse than that of REG and CC. For ${\hat{\mu }}_{2}$ , all specifications except for the IT approach with τ=1.7 achieved a lower RMSE than HAB with p=0.5 and were comparable to HAB with p=2 ; however, they remained less efficient than REG and CC.

Under balanced IPD, the DD RMSD cutoffs showed mixed performance. For ${\hat{\mu }}_{2}$ , the τ=1.7 OS specification maintained satisfactory bias across most conditions. The τ=2.7 specifications produced unsatisfactory negative bias when 30% of items drifted with δ=1.0 . The RMSE was primarily unsatisfactory for the τ=1.7 IT specifications, consistently exceeding 125 with I=20 items. For ${\hat{\sigma }}_{2}$ , the τ=1.7 specifications generally maintained satisfactory bias, whereas the τ=2.7 specifications showed unsatisfactory negative bias (up to −0.04 ) when 30% of the items drifted with δ=1.0 and I=40 items. The RMSE patterns mirrored those for the mean, with the IT approach showing lower efficiency. For ${\hat{\mu }}_{2}$ , the τ=1.7 OS specification performed similarly to CC, REG, and HAB ( p=2 ) in terms of both bias and RMSE. For RMSE, this also held for the τ=2.7 OS specification, although its bias lagged behind in the most demanding, balanced condition. For ${\hat{\sigma }}_{2}$ , bias was comparable to CC at τ=1.7 OS, but generally lower than REG and HAB, especially at p=2 , and RMSE remained lower than REG.

Under unbalanced IPD, the DD specifications did not maintain satisfactory performance in challenging conditions. For ${\hat{\mu }}_{2}$ , the τ=1.7 IT specification achieved satisfactory bias in select conditions with 10% or 30% IPD at larger sample sizes. Across specifications, positive bias increased with the percentage of IPD items and the effect size, δ . The RMSE was higher for the IT approach; the τ=1.7 IT specification reached 410 when 30% of the items drifted with δ=1.0 . For ${\hat{\sigma }}_{2}$ , negative bias was pervasive, reaching −0.18 for the τ=2.7 specifications under the severe IPD condition ( δ=1.0 and 30% IPD items) for I=20 and I=40 . The τ=1.7 IT specifications were the most variable, occasionally achieving satisfactory bias, although with extremely poor RMSE (up to 540). Compared to the reference methods, CC, REG, and HAB, all DD specifications performed worse in terms of both bias and RMSE for ${\hat{\mu }}_{2}$ and ${\hat{\sigma }}_{2}$ . One limited exception occurred for τ=1.7 , using the OS approach at larger N and 10% IPD, where the mean bias was comparable to that of HAB with p≤1 . However, RMSE still lagged behind REG and CC; τ=2.7 with OS yielded better RMSE, but at the cost of larger bias. For ${\hat{\sigma }}_{2}$ , both cutoffs showed more negative bias and higher RMSE than REG, CC, and HAB, especially p≥1 , including the severe condition ( δ=1.0 , 30% IPD items).

Overall, DD cutoffs yielded mixed results. Stricter cutoffs ( τ=1.7 ) with IT minimized bias in extreme conditions but sacrificed efficiency, particularly for I=20 items. The more lenient cutoff ( τ=2.7 ) maintained better efficiency, yet it failed to control bias under unbalanced IPD, especially at δ=1.0 and with 30% IPD items. These findings suggest that the optimal cutoff and approach choice depend critically on the expected pattern of IPD.

### Additional Results: Optimal Configuration of the Likelihood-Ratio Test

**Figure 3.**
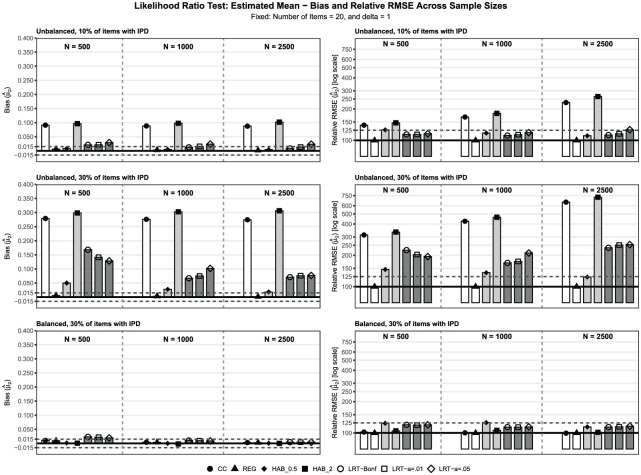
Bias and Relative RMSE for the Estimated Mean ${\hat{\mu }}_{2}$ in the Condition of I = 20 Number of Items, and IPD Effect Size δ = 1 as a Function of IPD Balance (Unbalanced, Balanced), Percentage of IPD Items (%IPD), and Sample Size N . *Note.* CC = concurrent calibration; REG = regularized linking; ${\mathrm{HAB}}_{0.5}$ = Haberman linking based on item intercepts with loss function power p = 0.5; ${\mathrm{HAB}}_{2}$ = Haberman linking based on item intercepts with loss function power p = 2; LRT-Bonf, LRT- α =.01, LRT- α =.05 = likelihood-ratio test applied to Haberman linking based on item difficulties with loss function power p = 2, using Bonferroni correction ( α = 0.05), α = 0.01, and α = 0.05, respectively.

Building on the main analysis, where the LRT with Bonferroni correction ( α=0.05 ) was selected in the most challenging condition (unbalanced IPD, 30% IPD, δ=1.0 , N=2500 , I=20 ), we examine whether this choice generalizes and how alternative significance levels perform. We consider three LRT specifications that all use the OS approach: α=0.05 , α=0.01 , and Bonferroni-corrected α=0.05/k . Re-estimation is kept fixed to HAB, with item difficulties and $L_{2}$ loss, as in the main analysis. As references, we report again CC, REG, and HAB, based on item intercepts with loss function powers p=.5 and p=2 . Performance is evaluated by bias and RMSE for the trend estimates of the mean ${\hat{\mu }}_{2}$ and the *SD* ${\hat{\sigma }}_{2}$ at T2. We show a subset of conditions for the estimated mean ${\hat{\mu }}_{2}$ in Figure 3 (unbalanced IPD with 10% and 30% drift, and balanced IPD with 30% drift, δ=1.0 , I=20 ). The complete tabular results for both ${\hat{\mu }}_{2}$ and ${\hat{\sigma }}_{2}$ are provided in the Supplement (Tables S15 to S20).

Under no IPD conditions, all LRT specifications maintain satisfactory bias for both trend parameters across most conditions. The Bonferroni correction shows a single instance with bias 0.02 for ${\hat{\mu }}_{2}$ , with I=40 and N=500 . RMSE values remain satisfactory across all specifications, although slightly elevated compared with the REG, particularly for smaller sample sizes.

Under balanced IPD, the LRT specifications show performance that varies with sample size. For ${\hat{\mu }}_{2}$ , all three significance levels produce unsatisfactory positive bias at N=500 across multiple conditions; values do not exceed 0.03 . As the sample size increases, bias decreases to satisfactory levels for most conditions at N=1,000 , and becomes consistently satisfactory at N=2,500 . RMSE values remain satisfactory in most conditions, with slight elevations at smaller sample sizes (notably at N=500 ). For ${\hat{\sigma }}_{2}$ , all LRT specifications maintain satisfactory bias across conditions. RMSE remains satisfactory overall, with slight elevations when 30% of items drift with δ=1.0 . Relative to CC, REG, and HAB, RMSE for ${\hat{\sigma }}_{2}$ under balanced IPD was generally higher for the LRT specifications.

Under unbalanced IPD, LRT specifications often fail to control bias adequately. For ${\hat{\mu }}_{2}$ , positive bias increases with both IPD percentage and effect size across all significance levels. The Bonferroni correction produces the highest bias, reaching 0.18 when 30% of items drift, with δ=1.0 . The α=0.01 and α=0.05 specifications exhibit similar patterns, but with slightly lower maximum bias values ( 0.14 and 0.13 , respectively). Bias decreases with increasing sample size, but remains unsatisfactory in most conditions. RMSE values exceed the 125 threshold in nearly all conditions with 30% IPD, reaching values above 300 at N=2,500 . For ${\hat{\sigma }}_{2}$ under unbalanced IPD, negative bias concentrates in the severe condition ( δ=1.0 and 30% IPD items) for both I=20 and I=40 . It is most pronounced for the Bonferroni correction and for α=0.01 at larger sample sizes. For α=0.05 , negative bias appears across all sample sizes in the 30% setting and is also present at δ=0.5 with 30% IPD items. RMSE becomes particularly poor when 30% of items drift, exceeding 200 in multiple conditions. Compared with CC, REG, and HAB, the RMSE for ${\hat{\sigma }}_{2}$ under unbalanced IPD was consistently worse for all LRT specifications, especially in the severe condition ( δ=1.0 , 30% IPD items).

The Bonferroni correction generally achieved the lowest bias and RMSE among LRT variants under moderate unbalanced IPD conditions, although all variants exceeded satisfactory thresholds in severe IPD conditions. The α=0.05 variant showed slightly better RMSE performance at δ=0.5 with 30% IPD (137 to 179) compared to the Bonferroni correction (154 to 200). The optimal significance level appears condition-dependent, where stricter corrections perform better under unbalanced IPD, while standard levels suffice for balanced conditions. The sample size dependency of performance demonstrated that the LRT requires large samples to function adequately, which limits its practical applicability, as is well known in the literature.

## Empirical Example

We illustrate the trend-estimation approaches using synthetic data derived from the ELFE reading comprehension test (Lenhard & Schneider, 2005), which was used in an earlier study by Robitzsch et al. (2011). We estimate the change from the first wave (T1) to the third wave (T2). The synthetic dataset was created using a data-augmented multiple-imputation approach (Grund et al., 2024; Jiang et al., 2022). This approach preserves the statistical properties of the original data while ensuring participant confidentiality. It combines partial least squares regression for dimension reduction with controlled noise injection (noise factor: 0.5 ) to generate synthetic observations that retain the original covariance structure and marginal distributions. Specifically, the approach preserves marginal distributions exactly while adding a calibrated amount of unreliability to protect individual responses (Grund et al., 2024; Jiang et al., 2022).

Before synthesis, the first item at T1 was removed because its p -value indicated insufficient fit. In addition, items T3I21 to T3I26 at T2 were excluded to prevent them from influencing the linking. Item labels range from I2 to I20, yielding 19 items total, where item 20 refers to label I20 rather than the twentieth sequential position. The dataset contains responses from N=1,855 students to I=19 reading comprehension items, administered at two time points: the end of Grade 3 (T1) and the end of Grade 4 (T2). All items are common across both time points. The R code for creating the synthetic data and reproducing this empirical example, along with the synthetic dataset, is available at https://osf.io/q86jz.

We apply the trend-estimation approaches examined in the simulation study and in the additional results to this two-time-point setting, using T1 as the reference scale with $\mu_{1}=\;0$ and $\sigma_{1}=1$ . Although the analysis focuses on two time points, the methods can readily be applied to additional time points via chain linking (e.g., Kolen & Brennan, 2014).

### Results

Table 6 presents the estimated distribution parameters for T2 across the trend-estimation approaches considered in the simulation study, as well as the additional results. For the mean at T2, estimates ranged from ${\hat{\mu }}_{2}=1.09$ (LRT with Bonferroni correction and re-estimation with HAB-b) to 1.57 (HAB-d with *p* = 0). CC and REG produced estimates of 1.26 and 1.25, respectively, while FC yielded an estimate of 1.20. Among the RMSD detection-based approaches, for FIX cutoffs, the estimates ranged from 1.22 to 1.32, depending on the cutoff and approach (OS vs. IT), while DD estimates ranged from 1.20 to 1.43. The LRT estimates varied with significance level, from 1.09 (Bonferroni) to 1.34 ( α=0.05 with HAE). The robust linking methods showed systematic variation based on the loss function parameter, with HAB-d estimates decreasing from 1.57 ( p=0 ) to 1.38 ( p=1 ), and a slight increase to 1.40 at p=2 , while HAE estimates remained around 1.20–1.22 across all p . *SD* estimates at T2 ranged from ${\hat{\sigma }}_{2}=0.95$ (DD with τ=2.7 , IT) to 1.38 (HAB-d with p=0 ). For CC, FC, and REG, the estimates were 1.14, 1.07, and 1.14, respectively. Within the detection-based approaches, RMSD with FIX cutoffs ranged from 1.06 to 1.20, DD from 0.95 to 1.13, and the LRT from 0.97 to 1.26, depending on the specification. The robust linking methods exhibited a decreasing trend with increasing p for HAB-d (from 1.38 to 1.20), whereas HAE remained stable between 0.96 and 0.98.

**Table 6.** Empirical Example: Estimated Trend Parameters for Direct Linking From T1 to T2 Using Different Trend-Estimation Approaches.

Method	${\hat{\mu }}_{2}$	${\hat{\sigma }}_{2}$
CC	1.26	1.14
FC	1.20	1.07
REG ( ε=0.001 )	1.25	1.14
LRT ( α=0.05 ) + HAB-b	1.29	1.26
LRT ( α=0.01 ) + HAB-b	1.16	1.19
LRT (Bonferroni) + HAB-b	1.09	1.21
LRT ( α=0.05 ) + ${\mathrm{HAE}}_{0.5}$	1.34	1.22
LRT ( α=0.01 ) + ${\mathrm{HAE}}_{0.5}$	1.26	1.06
LRT (Bonferroni) + ${\mathrm{HAE}}_{0.5}$	1.22	0.97
FIX (0.03, OS) + ${\mathrm{HAE}}_{1}$	1.32	1.20
FIX (0.03, IT) + ${\mathrm{HAE}}_{1}$	1.30	1.15
FIX (0.05, OS) + ${\mathrm{HAE}}_{1}$	1.22	1.06
FIX (0.05, IT) + HAE	1.22	1.06
FIX (0.08, OS) + ${\mathrm{HAE}}_{1}$	1.22	1.06
FIX (0.08, IT) + ${\mathrm{HAE}}_{1}$	1.32	1.06
DD ( τ=1.7 , OS) + ${\mathrm{HAE}}_{0.5}$	1.30	1.11
DD ( τ=1.7 , IT) + ${\mathrm{HAE}}_{0.5}$	1.43	1.13
DD ( τ=2.7 , OS) + ${\mathrm{HAE}}_{0.5}$	1.20	0.95
DD ( τ=2.7 , IT) + ${\mathrm{HAE}}_{0.5}$	1.21	0.98
HAB-d ( p=0 )	1.57	1.38
HAB-d ( p=.25 )	1.51	1.32
HAB-d ( p=.5 )	1.44	1.27
HAB-d ( p=1 )	1.38	1.21
HAB-d ( p=2 )	1.40	1.20
${\mathrm{HAE}}_{0.5}$ ( p=0 )	1.21	0.97
${\mathrm{HAE}}_{0.5}$ ( p=.25 )	1.22	0.98
${\mathrm{HAE}}_{0.5}$ ( p=.5 )	1.21	0.98
${\mathrm{HAE}}_{0.5}$ ( p=1 )	1.21	0.97
${\mathrm{HAE}}_{0.5}$ ( p=2 )	1.20	0.96

*Note.* CC = concurrent calibration; FC = fixed calibration; REG = regularized estimation using smooth Bayesian information criterion with tuning parameter ε ; LRT = likelihood-ratio test with significance level α (Bonferroni = α=0.05/k ); FIX = root mean square deviation with fixed cutoff; DD = root mean square deviation with data-driven cutoff threshold τ ; OS = one-step approach; IT = iterative approach; HAB-b = Haberman linking based on item difficulties $b_{i}$ ; HAB-d = Haberman linking based on item intercepts $d_{i}$ ; ${\mathrm{HAE}}_{0.5}$ = Haebara linking with normal-density weighting ( σ=0.5 ); ${\mathrm{HAE}}_{1}$ = Haebara linking with normal-density weighting ( σ=1 ); p = power of the $L_{p}$ loss function. For LRT, FIX, and DD methods, trends are re-estimated under partial invariance using the specified linking method after detection of drifting items.

The detection-based methods identified varying numbers of items with IPD, resulting in different anchor sets. The LRT approaches flagged between 4 and 12 items depending on the significance level: Bonferroni correction identified Items 5, 9, 14, and 18; α=.01 identified Items 2, 4, 5, 9, 14, 18, and 20; and α=.05 identified Items 2–5, 9, 11–14, 18, 19, and 20. The FIX cutoff RMSD method showed varying sensitivity across cutoff values and detection approaches: the 0.03 cutoff, the OS approach flagged 15 (3–14, 18–20), while the IT approach flagged 16 items (2–14, 18–20). In contrast, both the 0.05 and 0.08 cutoffs detected no items under either approach. The DD RMSD method with τ=1.7 identified 10 items (2, 7, 10, 11, 15–20) using the OS approach and 11 items (2, 7, 10, 11, 14–20) using the IT approach. The τ=2.7 cutoff detected only item 15 with the OS approach, whereas the IT approach identified three items (15, 16, and 20). The regularization approach identified only item 9 as having drift ( ${\hat{\delta }}_{9}=-0.5$ ), with the sum of all IPD effects being nonzero, indicating unbalanced IPD.

The difference of 0.48 between the lowest and highest mean estimates could lead to different conclusions about trends. If the true trend is 1.09, as suggested by LRT with Bonferroni correction, this indicates progress of approximately one *SD* over the school year. If the true growth is 1.57, as with HAB-d at p=0 , this represents a trend exceeding 1.5 *SD*. The variation in *SD* estimates (0.95 to 1.38) likewise affects interpretations of variance in student growth.

## Discussion

This article investigated trend-estimation approaches across two time points under sparse, uniform IPD in the 2PL model. A comparative analysis was conducted of five approaches for trend estimation: CC, FC, robust linking with HAB and HAE, partial invariance using LRT and RMSD with FIX and DD cutoffs, and REG with the SBIC.

In the absence of IPD, nearly all methods performed satisfactorily across conditions, consistent with previous findings demonstrating optimal performance of CC and FC under correct model assumptions (Hanson & Béguin, 2002; Jodoin et al., 2003; S.-H. Kim & Cohen, 1998; Kolen & Brennan, 2014; Robitzsch & Lüdtke, 2022).

In balanced IPD settings, CC remained unbiased and efficient for ${\hat{\mu }}_{2}$ . However, this was not the case for ${\hat{\sigma }}_{2}$ , particularly for an IPD effect size δ=1.0 , aligning with prior research showing that balanced IPD can still negatively affect *SD* estimation in the 2PL model (Robitzsch, 2023a; Robitzsch & Lüdtke, 2022). Robust linking with p≥1 maintained good efficiency (He & Cui, 2019; Robitzsch, 2023a), while small p values reduced bias marginally but increased variance in shorter tests. The RMSD with DD cutoffs and other detection-based variants sometimes exhibited efficiency losses for I=20 , while achieving acceptable bias control in many balanced conditions. Furthermore, under balanced IPD, HAB performed acceptably with p=2 and outperformed the other loss functions. This pattern did not hold for HAE at p=2 , which deteriorated under 30% IPD, δ=1.0 , with unsatisfactory negative bias and elevated RMSE for both trend parameters. However, it did not vary in its efficiency across loss functions for $\mu_{2}$ , unlike HAB.

Under conditions of unbalanced IPD, CC and FC exhibited bias across all conditions, confirming earlier findings that unbalanced IPD introduces substantial bias in methods that assume full invariance (DeMars, 2019; Robitzsch, 2023a). This bias increased with increasing δ and the percentage of IPD items. The RMSD with DD cutoffs mitigated bias in severe unbalanced settings, but this occurred at the expense of efficiency for the smaller number of items, I=20 . Under unbalanced IPD conditions, CC and FC exhibited bias in the estimated mean, with values reaching 0.28 for CC and 0.21 for FC in the most severe conditions. Regarding robust linking, lower loss function powers ( p≤1 ) effectively controlled bias under unbalanced IPD, with HAB using p=0 showing bias in only three conditions. In comparison, higher powers (*p*= 2) yielded better efficiency under no IPD but severe bias under unbalanced conditions, extending previous findings on the trade-off between robustness and efficiency (Robitzsch, 2020; Robitzsch & Lüdtke, 2022). In addition, a smaller mean shift at T2 ( $\mu_{2}=0.5$ ) was considered, and the pattern of findings was found to be similar to the main results (see Tables S21–S26 in the Supplement).

Overall, the regularization approach using SBIC proved to be the most consistently effective across conditions, and our findings corroborate Robitzsch (2024b) by demonstrating that REG using SBIC provides satisfactory parameter recovery under no or balanced IPD. Extending the 40% contamination design, we found that regularization maintained acceptable performance at 10% and 30% drift rates across various sample sizes. Under unbalanced IPD, robust HAB with p=0 achieved in more conditions a satisfactory bias than regularization for ${\hat{\mu }}_{2}$ , but with some efficiency loss. Notably, REG remained within acceptable limits at the largest sample size ( N=2,500 ), suggesting its viability for adequately powered studies. The RMSD with DD cutoffs demonstrated potential, but its inefficiency for I=20 was a notable limitation that stemmed from the IT approach.

### Limitations and Future Research

As with any simulation, the conclusions are bounded by the particular conditions investigated. We assumed that the scaling model, the 2PL model, was specified correctly. However, it is important to note that data may also be generated by more complex or multidimensional item-response models. Therefore, it is advisable to exercise caution when generalizing to contexts characterized by distinct or as yet unidentified data-generating processes. Evaluating the primary specifications under alternative IRT models, such as the 1PL and 3PL, would provide additional insight, given their practical use (Arce-Ferrer & Bulut, 2017; Fischer et al., 2021; Huggins, 2014). Future research could also investigate the trend-estimation approaches under model misspecification (Bolt et al., 2014; Fischer et al., 2021; Samejima, 2000; Xu et al., 2009), as well as guessing and slipping (Culpepper, 2017; DeMars & Jurich, 2015). Moreover, this study used only dichotomous items. Extending the design to polytomous items or mixed-format data would provide valuable insights (Andersson, 2018; S. Wang et al., 2024; Zhao & Hambleton, 2017). The exploration of nonuniform sparse IPD in item discriminations could be another area for future research. In the current study, which investigated uniform IPD, regularization was specified for the data-generating model, with a penalty term only applied to the item intercept IPD effects. In contrast, HAB applied robust loss functions for both the mean and *SD*. However, uniform IPD does not affect the estimation of *SD* under HAB, and LRT tests both intercepts and discriminations. Under uniform IPD, HAB could alternatively be applied with constrained linking, which sets discrimination parameters invariant while allowing non-invariant intercepts (Chen et al., 2023). This approach would likely improve its performance in terms of bias and RMSE, approaching the results achieved by regularization. Under nonuniform IPD conditions, regularization could be enhanced by incorporating a second set of IPD effects for the item discriminations (see Schauberger & Mair, 2020), and the relative performance compared to HAB, as applied here, should be investigated. In addition, the five trend-estimation approaches could be examined under chain and joint linking involving three or more time points, as well as under different longitudinal linking designs (e.g., booklet, consecutive time points with adjacent items, or common items across all time points) (e.g., Battauz, 2013; Engels et al., 2025; Keller & Keller, 2011). Investigating proportions of IPD items greater than 30% (not exceeding 50%, see Halpin, 2024; W. Wang et al., 2022) presents another promising avenue for future inquiry. It is important to note that applications may contain fewer common items than those considered here. Therefore, examining smaller sets, such as I=15 or fewer (Robitzsch, 2025b), would be beneficial. Future research should also consider the incorporation of unique items and their effects on various trend-estimation approaches (Engels et al., 2025). In addition, the impact of unequal sample sizes on trend-estimation methods could be addressed in future studies, as this often poses a challenge in longitudinal designs when participants drop out (Cho et al., 2016; DeMars, 2019; Woods, 2008). Unequal group sizes also arise when linking large item banks or multiple measurement occasions, not only in simple two-group comparisons. The forward-only IT approach was chosen for computational feasibility in this study. LRT could also be applied with item purification, and FC could be used as another re-estimation method under partial invariance (González-Betanzos & Abad, 2012; König et al., 2021; W.-C. Wang & Yeh, 2003). A variety of IT approaches have been developed, including approaches that allow the re-evaluation of previously flagged items or the use of different anchor sets, which could be compared with the trend-estimation methods employed in this study in future studies (Kopf et al., 2015b). A small dedicated simulation comparing OS and IT approaches, including computational efficiency considerations, would provide valuable guidance for practitioners.

The article operated under the assumption that IPD was sparse and construct-irrelevant (Camilli, 1993; El Masri & Andrich, 2020; Shealy & Stout, 1993), necessitating statistical adjustments or item removal (e.g., Halpin, 2024; Magis & De Boeck, 2011; Robitzsch, 2023a). This approach effectively removes drifting items from the measurement of change over time. However, an alternative perspective posits that IPD may be construct-relevant, reflecting meaningful changes in the latent construct itself. Under this framework, adjusting for IPD could compromise construct validity by narrowing the scope of what is measured (Camilli, 1993; El Masri & Andrich, 2020; Han et al., 2012; Robitzsch & Lüdtke, 2022).

## Conclusion

In instances where unbalanced IPD is suspected, methods that down-weight or shrink IPD, such as HAB with p=0 or REG with SBIC, are preferable to approaches that assume full invariance (CC and FC) or to HAE. For large sample sizes ( N=2,500 ), REG with SBIC proved to be the optimal method across all conditions. The trade-offs associated with partial invariance using IPD statistics should be considered. While these methods can achieve satisfactory outcomes, their efficacy depends on the appropriate selection of cutoffs, which may not be known in practical settings. The choice of cutoffs is not trivial, as different thresholds can lead to different conclusions about the magnitude of the trend. In the context of robust linking, non-robust p=2 specifications were most efficient when IPD was absent or balanced. Conversely, smaller p values have been shown to enhance robustness under conditions of unbalanced IPD. Given the trade-offs between different loss function powers, when the nature of potential IPD is unknown, intermediate values such as p=.5 or p=1 appear to offer reasonable compromises between efficiency and robustness, as indicated by this study. Due to the superior efficiency of REG with SBIC over HAB and RMSD with DD cutoffs, it can be recommended that practitioners prioritize regularized estimation when computational resources permit and sample sizes are adequate.
